# Enhancing radiotherapy response via intratumoral injection of a TLR9 agonist in autochthonous murine sarcomas

**DOI:** 10.1172/jci.insight.178767

**Published:** 2024-06-13

**Authors:** Chang Su, Collin L. Kent, Matthew Pierpoint, Warren Floyd, Lixia Luo, Nerissa T. Williams, Yan Ma, Brian Peng, Alexander L. Lazarides, Ajay Subramanian, Jonathon E. Himes, Vincent M. Perez, Rosa D. Hernansaiz-Ballesteros, Kimberly E. Roche, Jennifer L. Modliszewski, Sara R. Selitsky, Mari L. Shinohara, Amy J. Wisdom, Everett J. Moding, Yvonne M. Mowery, David G. Kirsch

**Affiliations:** 1 Department of Pharmacology and Cancer Biology and; 2 Department of Radiation Oncology, Duke University Medical Center, Durham, North Carolina, USA.; 3 Department of Radiation Oncology, MD Anderson Cancer Center, Houston, Texas, USA.; 4 Department of Radiation Oncology, Stanford University, Stanford, California, USA.; 5 Tempus AI Inc., Durham, North Carolina, USA.; 6 QuantBio LLC, Durham, North Carolina, USA.; 7 Lineberger Comprehensive Cancer Center, Chapel Hill, North Carolina, USA.; 8 Department of Integrative Immunology,; 9 Department of Molecular Genetics and Microbiology, and; 10 Department of Neurobiology, Duke University School of Medicine, Durham, North Carolina, USA.; 11 Harvard Radiation Oncology Program, Boston, Massachusetts, USA.; 12 Stanford Cancer Institute, Stanford University, Stanford, California, USA.; 13 Department of Radiation Oncology, University of Pittsburgh, Pittsburgh, Pennsylvania, USA.; 14 Department of Radiation Oncology and; 15 Department of Medical Biophysics, University of Toronto, Toronto, Ontario, Canada.; 16 Radiation Medicine Program, Princess Margaret Cancer Centre, University Health Network, Toronto, Ontario, Canada.

**Keywords:** Immunology, Oncology, Cancer immunotherapy, Radiation therapy, T cells

## Abstract

Radiation therapy (RT) is frequently used to treat cancers, including soft-tissue sarcomas. Prior studies established that the toll-like receptor 9 (TLR9) agonist cytosine-phosphate-guanine oligodeoxynucleotide (CpG) enhances the response to RT in transplanted tumors, but the mechanisms of this enhancement remain unclear. Here, we used CRISPR/Cas9 and the chemical carcinogen 3-methylcholanthrene (MCA) to generate autochthonous soft-tissue sarcomas with high tumor mutation burden. Treatment with a single fraction of 20 Gy RT and 2 doses of CpG significantly enhanced tumor response, which was abrogated by genetic or immunodepletion of CD8^+^ T cells. To characterize the immune response to CpG+RT, we performed bulk RNA-Seq, single-cell RNA-Seq, and mass cytometry. Sarcomas treated with 20 Gy and CpG demonstrated increased CD8 T cells expressing markers associated with activation and proliferation, such as Granzyme B, Ki-67, and IFN-γ. CpG+RT also upregulated antigen presentation pathways on myeloid cells. Furthermore, in sarcomas treated with CpG+RT, TCR clonality analysis suggests an increase in clonal T cell dominance. Collectively, these findings demonstrate that CpG+RT significantly delays tumor growth in a CD8 T cell–dependent manner. These results provide a strong rationale for clinical trials evaluating CpG or other TLR9 agonists with RT in patients with soft-tissue sarcoma.

## Abstract 2

Combining radiation therapy with TLR9 agonist CpG delays tumor growth in soft tissue sarcomas through a CD8^+^ T cell–dependent mechanism.

## Graphical abstract

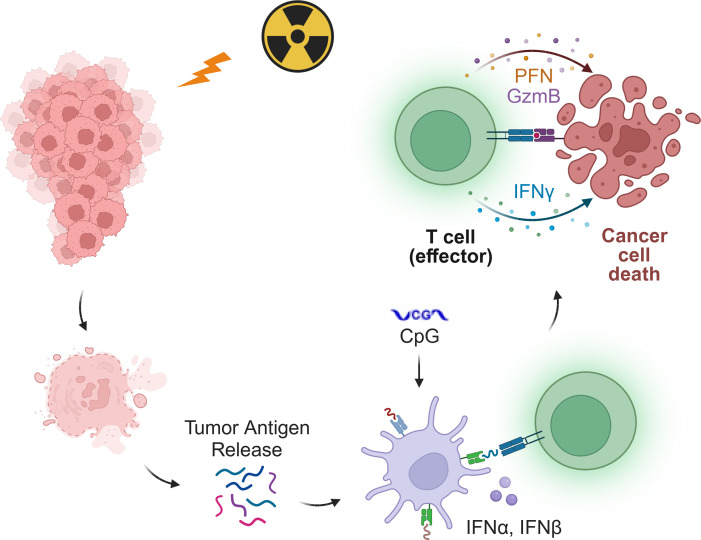

## Introduction

Soft-tissue sarcomas (STS) are a heterogeneous group of malignancies (1). Following local therapies with surgery and radiotherapy, approximately 50% of patients with large, high-grade tumors develop distant metastasis. After metastases occur, limited therapeutic options are available, with median survival of approximately 12–18 months (2). Thus, there is a pressing need for alternative therapeutic approaches to improve overall survival for these patients.

Immunotherapy has recently emerged as a promising treatment for many solid tumors (3), with particularly high response rates in melanoma and non–small cell lung carcinoma (NSCLC). However, a phase II clinical trial in patients with advanced STS, SARC028, showed that only ~17.5% of patients responded to anti–PD-1 monotherapy, which suggests that sarcomas are more resistant to immune checkpoint blockade compared with 40%–45% objective response rates in patients with melanoma and NSCLC (4). Nevertheless, it should be noted that there are more than 50 histological subtypes for STS, each with its own distinct biological characteristics (5, 6). STS subtypes respond to various therapies differently. For instance, undifferentiated pleomorphic sarcoma (UPS) showed a more favorable response to pembrolizumab compared with other subtypes of sarcoma in SARC028 (4). Therefore, clinical trials of immunotherapy that enroll patients with multiple subtypes of sarcomas may underestimate the effect of immunotherapy in the most responsive subtypes of sarcoma.

Over 40 years ago, Stone and colleagues used a transplanted model of STS to discover that activating the immune system through bacterial infection can enhance tumor control when administered with radiation therapy (RT) (7). Many subsequent studies have suggested that RT can work synergistically with immunotherapy to suppress tumor growth (3, 8, 9). In this study, we found that the combination of CpG (unmethylated cytosine-phosphorothioate-guanosine forms of DNA), a TLR9 agonist, and RT suppresses tumor growth significantly using autochthonous mouse models of STS in which the tumor gradually develops under surveillance by an intact immune system (10, 11). Here, we show that CD8^+^ T cells are essential for mediating the antitumor effects of CpG and radiotherapy. We further demonstrate that depleting lymphocytes, especially CD8^+^ T cells, negate the treatment effects of CpG. Unlike immune checkpoint inhibitors that aim to reverse the exhaustion state of T cells, the TLR9 agonist CpG combined with RT draws CD8^+^ T cells expressing markers associated with activation and proliferation into the tumor. Taken together, these findings suggest a promising treatment option of combining TLR9 agonists and radiotherapy in treating patients with STS, a condition that often contains few T cells.

## Results

### CpG and RT suppress autochthonous p53/MCA sarcoma growth.

**Figure 1:**
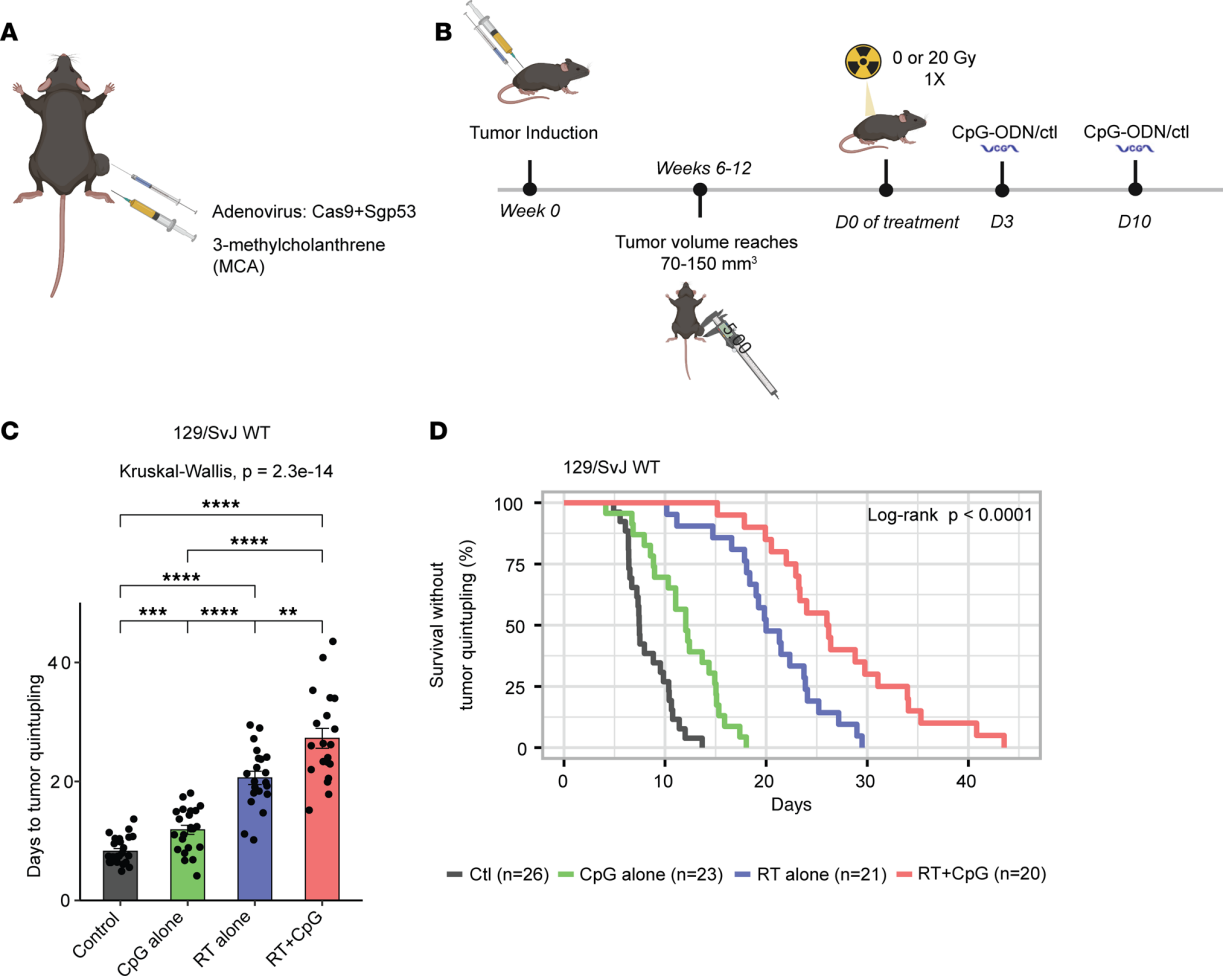
Increased tumor growth delay after treatment with CpG ODN and radiation therapy in autochthonous p53/MCA sarcomas. (**A**) Primary sarcoma initiation by intramuscular injection of Adeno-Cas9-sgp53 and MCA. (**B**)Autochthonous sarcomas develop at the injection site about 7–11 weeks after injection. Mice were treated with CpG ODN or control GpC dinucleotides and 0 or 20 Gy when tumors reached > 70 mm^3^. (**C**) Mice with p53/MCA sarcomas received control GpC dinucleotides with 0 Gy (black, *n* = 26), CpG ODN alone (green, *n* = 23), control GpC dinucleotides with 20 Gy (blue, *n* = 21), or CpG ODN with 20 Gy (red, *n* = 20). Time to tumor quintupling (days) after the indicated treatment. (**D**) Mice with p53/MCA sarcomas received control GpC dinucleotides with 0 Gy (black, *n* = 26), CpG ODN alone (green, *n* = 23), control GpC dinucleotides with 20 Gy (blue, *n* = 21), or CpG ODN with 20 Gy (red, *n* = 20). Kaplan-Meier analysis with tumor quintupling as the endpoint. Kruskal-Wallis test was used for comparison across the groups, while the Wilcoxon test was selected for the pair-wise comparisons. **P ≤ 0.01, ****P ≤ 0.0001.

To investigate whether the combination treatment of CpG and RT improves tumor growth delay compared with single therapy with CpG alone or RT alone in a primary tumor model, we induced STS with a high mutational load in 129/SvJae mice by injecting an adenovirus-expressing CRISPR-Cas9, a single-guide RNA (sgRNA) targeting *Trp53* (sgp53) (10), and the carcinogen 3-methylcholanthrene (MCA) into the gastrocnemius muscle (11) (Figure 1A). After tumor induction, primary sarcomas (p53/MCA model) develop at the injection site over 2–3 months under the selective pressure of immunoediting in immunocompetent mice (12). p53/MCA sarcomas demonstrate gross and histologic morphologies as well as transcriptional profiles similar to human UPS (10, 13). When tumor volumes reached 70–150 mm^3^, mice were randomized to receive 0 Gy or 20 Gy RT (Day 0 [D0]) and CpG or control (GpC dinucleotides with the positions of cytosine and guanine reversed relative to the phosphate linker) (D3 and D10) (Figure 1B). Significant tumor growth delay was observed with either CpG alone or RT alone compared with the control group. Mice treated with CpG and RT exhibited the longest time to tumor quintupling with a mean of 27.2 days compared with 8.3 days for mice treated with control, 11.9 days for CpG alone, and 20.6 days for radiotherapy alone, suggesting that the TLR9 agonist improves radiotherapy’s treatment effect in delaying tumor growth (Figure 1, C and D).

### CyTOF demonstrates significantly increased activated CD8^+^ T cells in CpG+RT-treated tumors.

**Figure 2:**
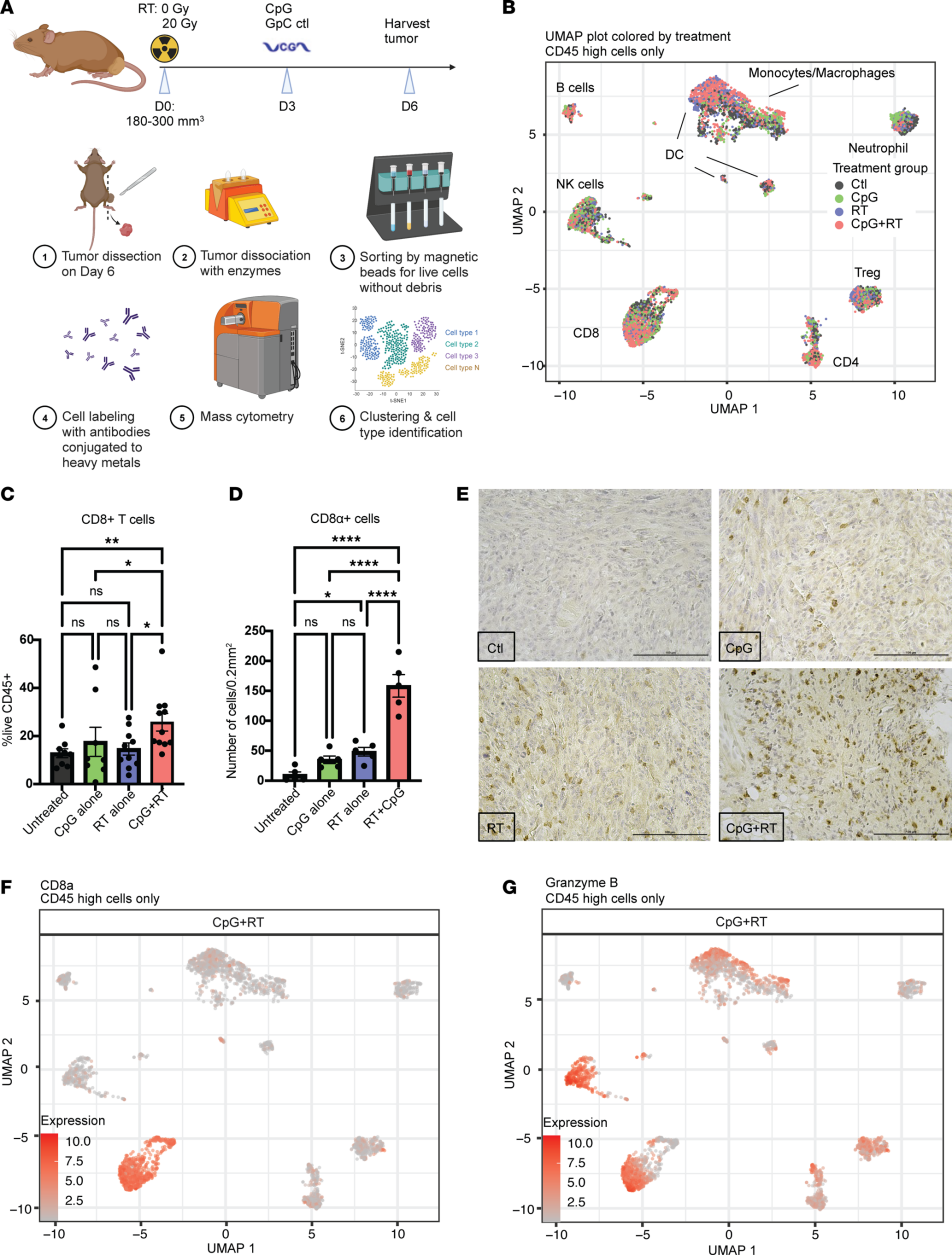
CyTOF and IHC staining demonstrates enhanced intratumoral infiltration of activated CD8 T cells after combination treatment. (**A**) Treatment schedule and tumor processing schematic. (**B**) UMAP plot of CyTOF data clustering for all CD45^hi^ cells from all tumors and treatment groups. (**C**) Frequency of CD8^+^ cells/live CD45^+^ cells by CyTOF. Data show mean ± SEM, analyzed by 3-way ANOVA. (**D**) Average number of CD8^+^ cells/0.2 mm^2^ in IHC slides.(**E**)Representative IHC staining with CD8 Ab. Scale bar: 100 μm. (**F**) CD8 expression in CyTOF CD45 high UMAP plot. (**G**) Granzyme B expression in CyTOF CD45^hi^ UMAP plot. *P ≤ 0.05, **P ≤ 0.01, ****P ≤ 0.0001.

To begin to investigate if CpG is mediating growth delay by acting on immune cells rather than killing tumor cells directly, we performed an IncuCyte Live-Cell assay to monitor cell proliferation of 3 p53/MCA tumor cell lines after coincubation with titrated concentrations of CpG. The in vitro assay demonstrated that CpG does not directly inhibit the proliferation of p53/MCA tumor cell lines (Supplemental Figure 1; supplemental material available online with this article; https://doi.org/10.1172/jci.insight.178767DS1), indicating that in vivo tumor growth delay induced by CpG alone is not through direct tumor cell killing. To determine which cell populations play an important role in mediating the treatment effects of CpG and RT, we performed mass cytometry (CyTOF) on tumor cells and tumor-infiltrating immune cells (Figure 2A) (14). We induced p53/MCA tumors as described above and initiated treatment when tumors reached 180–300 mm^3^. We investigated the tumor immune microenvironment on D3 after CpG (D6 after RT) because we typically observed prominent tumor shrinkage at this time point in mice receiving combination therapy. Figure 2B shows a Uniform Manifold Approximation and Projection (UMAP) plot of CD45^+^ cells characterized by CyTOF for the 4 treatment groups. We observed significantly more CD8^+^ T cells in tumors from mice treated with CpG and RT (Figure 2C) compared with the other treatment groups, which was confirmed by CD8 IHC staining (Figure 2, D and E). These CD8^+^ T cells coexpressed Granzyme B and Ki-67 (*R* = 0.36, *P* ≤ 0.0001), indicating that these CD8^+^ T cells are not exhausted but rather are actively proliferating and primed for cytotoxic cell killing (Figure 2, F and G, and Supplemental Figure 2). CpG alone or RT alone does not increase CD8^+^ T cells significantly with CyTOF analysis, but RT alone does draw significantly more CD8^+^ T cells to the intratumoral area when compared with untreated tumors by IHC staining. Nevertheless, CpG+RT draws a significantly higher number of CD8^+^ T cells to the intratumoral area when compared with other treatment groups.

### Single-cell RNA-Seq reveals changes in the adaptive immune system after CpG+RT combination therapy.

**Figure 3:**
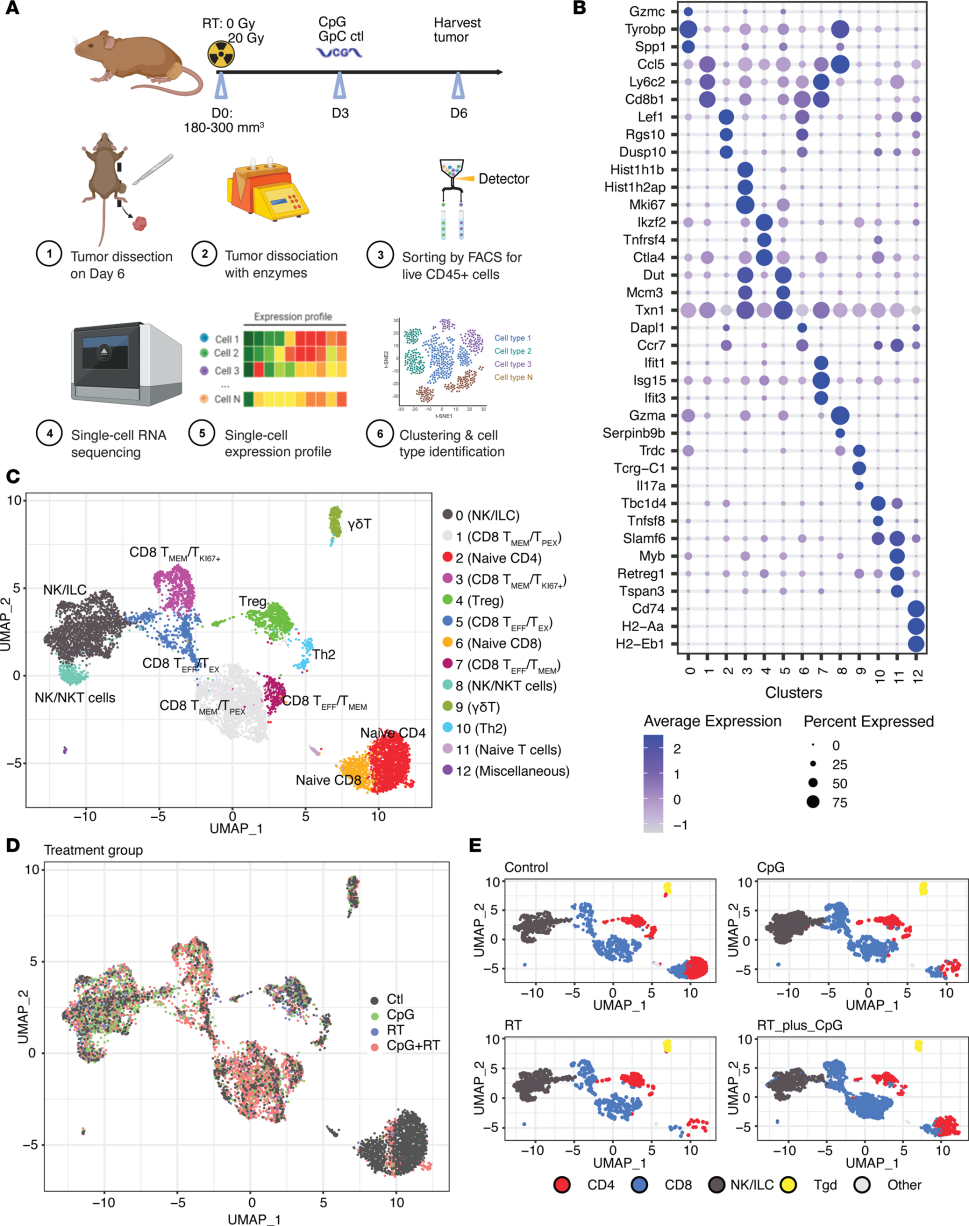
scRNA-Seq shows increased CD8 T cell infiltration into the tumor after CpG+RT. (**A**) Treatment schedule and tumor processing schematic. (**B**) Bubble plot of top 3 differentially expressed genes in each of the T cell subclusters. The shades of color are correlated with levels of expression. The sizes of circles are correlated with percentage of cells in that cluster that express the gene of interest. (**C**) UMAP plot of T cell and NK cell scRNA-Seq subclustering. (**D**) UMAP plot of T cell and NK cell subclustering colored by treatment groups.(**E**) UMAP plot of lymphocytes subclustering colored by CD4 (red), CD8 (blue), NK/ILC (black), and γδT (yellow) cells. Mice with p53/MCA sarcomas received control GpC dinucleotides with 0 Gy (*n* = 5), CpG ODN alone (*n* = 5), control GpC dinucleotides with 20 Gy (*n* = 5), or CpG ODN with 20 Gy (*n* = 5).

To identify the major transcriptional differences in immune cell populations with and without CpG and RT, we performed single-cell RNA-Seq (scRNA-Seq) on CD45^+^ tumor-infiltrating immune cells from an independent cohort of sarcomas sorted by FACS and harvested 6 days after 0 or 20 Gy RT and 3 days after Control or CpG (Figure 3A). After filtering and quality control, scRNA-Seq analysis generated data for 94,093 cells. Unbiased clustering using shared nearest neighbor (SNN) modularity optimization identified 20 cell clusters with distinct transcriptional profiles that were assigned to known cell lineages utilizing Seurat (15) (Supplemental Figure 3A). The predominant cell populations of intratumoral immune cells were myeloid cells, and this is consistent with previous scRNA-Seq analyses for primary p53/MCA sarcomas (12).

Since CD8^+^ T cells exhibited the most significant increase in cell numbers based on CyTOF, we subclustered T cells and NK cells to better identify transcriptional differences among sarcomas from the 4 treatment groups. Unsupervised clustering analysis resulted in the identification of 13 subpopulations. The 13 clusters were further annotated based on differentially expressed genes (DEGs) and canonical immune markers, including NK cells/innate lymphoid cells/NKT cells (c0 and c8), CD4^+^ T cells (c2, c9, and c10), Treg (c4), and CD8^+^ T cells (c1, c3, c5, c6, and c7) (Figure 3, B and C, and Supplemental Table 1). The most prominent difference across treatment groups was found in the CpG+RT treatment group with a higher number of activated CD8^+^ T cells (c1, c3, c5, c7) that express elevated levels of Granzyme B (*Gzmb*), Granzyme K (*Gzmk*), and IFN-γ (*Ifng*) (Figure 3, D and E; Supplemental Figure 3, B and C; and Supplemental Figures 4 and 5). Cell cycle distribution for these clusters is shown in Supplemental Figure 3D, which demonstrates that the majority of the CD8^+^ T cells are actively proliferating and not yet exhausted.

The majority of CD8^+^ T cells in the combination treatment group clustered to c1, c3, c5, and c7. Cluster c1 expresses high levels of *Ccl5*, *Ly6c2*, *Cxcr6*, *Cd28*, *Gzmk*, *Ifng*, *Tnfaip3*, and *Tnfaip8* (Figure 3B and Supplemental Figure 3B). *Ccl5* and *Ly6c2* are highly associated with the maintenance and homing of memory T cells (16–18). Connolly and colleagues also identified a group of memory CD8^+^ T cells expressing high levels of *Ccl5* and *Ly6c2* in C57BL/6 mice that were infected with acute lymphocytic choriomeningitis virus (LCMV) (18). *Cxcr6* is a marker for resident memory T cells and antitumor efficacy (19–21). *Cd28*, *Gzmk*, *Ifng*, *Tnfaip3*, and *Tnfaip8* are activation markers. However, roughly half of the cells in c1 also express exhaustion markers such as *Pdcd1* and *Ctla4* (18, 22). Therefore, this population is likely a group of effector memory T cells, with some transitioning into early exhaustion. Furthermore, c3 is comprised of CD8^+^ T cells that express genes associated with cell proliferation, such as *Mki67*, *Top2a*, and *Birc5* (23, 24) (Figure 3B and Supplemental Figure 3B), and it also expresses high levels of *Runx3*, *Gzmb*, with a small percentage of cells expressing *Pdcd1* and *Ctla4*. *Runx3* is a key regulator of tissue-resident memory CD8^+^ T cell differentiation and homeostasis, while the other genes are associated with effector and early exhaustion CD8^+^ T cells (25). Cluster c5 is another cluster of CD8^+^ T cells that also express high levels of *Runx3*, *Cdca7*, *Stmn1*, *Txn1*, *Ifng*, and granzyme-related genes (Figure 3B and Supplemental Figure 3B). These genes are associated with higher immune cell infiltration, as well as proliferative CD8^+^ T cells (26–30). However, about 50% of the cells in c5 also express inhibitory receptors and transcription factors such as *Pdcd1* and *Ctla4*, which indicate that some of these cells are transitioning into an exhausted state (18). Cluster c7 expresses *Ifng*, *Gzmk*, *Gzmb*, and *Ly6c2*, suggesting that this cluster contains mostly effector memory T cells (31) (Figure 3B and Supplemental Figure 3B). Taken together, these data illustrate that there are increased effector/memory CD8^+^ T cells in the tumor after combination treatment with CpG and RT. In addition, these CD8^+^ T cells express relatively low levels of genes associated with exhaustion; usually less than 50% of T cells within a cluster express *Pdcd1* and *Ctla4*, which are also usually upregulated in activated T cells. Other common genes associated with exhaustion, such as *Lag3*, *Havcr2*, and *Tox*, are hardly expressed in these intratumoral T cells (Supplemental Figure 3B) (22). These T cells are also highly proliferative, indicating that CpG+RT attracts CD8^+^ T cells, which are not yet exhausted, into the tumors.

The population sizes of NK/ILC/NKT cells (c0 and c8 expressing *Ncr1*), Tregs (c4 expressing *Foxp3*), γδT cells (c9 expressing *Tcrg-C1*), Th2 cells (c10 expressing *Tbc1d4* and *Tnfsf8*), and a population of myeloid cells (c12 expressing *Cd74* and *H2-Aa*) did not differ significantly between the 4 treatment groups (Figure 3B and Supplemental Figure 3, B and C) (30, 32–35). The 3 populations that were lower after CpG+RT compared with the control group are c2, c6, and c11 (Supplemental Figure 3C). Cluster c2 differentially expresses *Cd4*, *Lef1*, *S1pr1*, and *Ccr7*, which are genes generally associated with naive CD4^+^ T cells (36–40). Cluster c6 expresses *Cd8a*, *Lef1*, *Ccr7*, and *Il7r*, which are usually associated with naive CD8^+^ T cells (41). Cluster c11 is a cluster with very few cells that expresses *Slamf6*, *Eomes*, and *Foxp1*, which are often associated with exhausted or naive T cells (Figure 3B and Supplemental Figure 3B). Taken together, these data indicate that CpG+RT resulted in increased infiltration of CD8^+^ T cells, with these cells displaying a range of transcriptional profiles indicative of effector memory, proliferation, and early exhaustion states. However, naive CD4^+^ and CD8^+^ T cells were more abundant in untreated tumors, suggesting a shift in the immune landscape with CpG+RT. Both CpG-treated and CpG+RT-treated tumors express higher levels of *Ifng* and *Gzmb* when compared with control. Tumors treated with RT alone also express higher levels of *Gzmb* when compared with control. However, CpG+RT upregulated the transcription of *CD8*, *Ifng*, and *Gzmk* to a greater extent when compared with tumors in the other treatment groups (Supplemental Figures 4 and 5). This is consistent with the CyTOF and IHC staining results.

### Bulk RNA-Seq demonstrated a similar increase in CD8^+^ T cells after CpG+RT treatment and specific T cell clonal expansion.

To gain insight into the overall transcriptional differences in the tumor and immune microenvironments, we performed bulk tumor RNA-Seq on an independent cohort of p53/MCA tumors harvested 6 days after RT and 3 days after CpG treatment or their respective controls. We then performed digital cytometry on the bulk tumor RNA-Seq data set using CIBERSORTx to estimate the abundance of 22 different immune cell populations in the tumor microenvironment (42). CIBERSORTx results from the bulk tumor RNA-Seq data set support the findings from CyTOF, CD8 IHC, and scRNA-Seq that CD8^+^ T cells increase significantly after treatment with CpG and RT (Figure 2C; Figure 3, D and E). These results from multiple orthogonal assays are consistent with the hypothesis that the superior treatment effect of combination treatment is mediated through influx, activation, and proliferation of CD8^+^ T cells.

**Figure 4:**
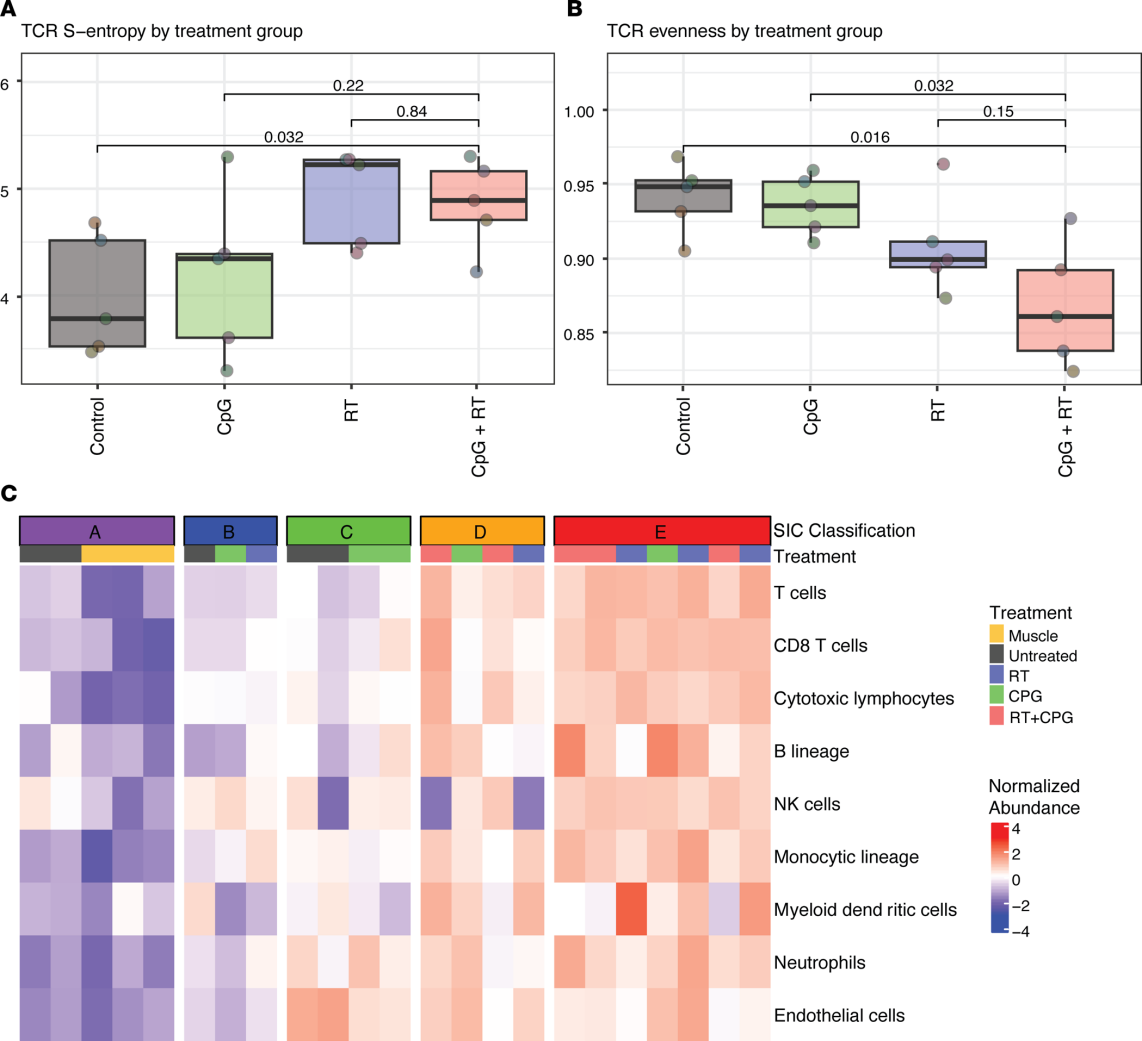
Treatment with CpG+RT promotes tumor-antigen–specific clonal expansion of T cells and tumor immune microenvironment remodeling. (**A**) p53/MCA sarcoma develops at the injection site about 7–11 weeks after induction. Mice were treated with CpG ODN or GpC dinulcotides control and 0 or 20 Gy when tumors reached > 180 mm^3^. Sarcomas received control GpC dinucleotides with 0 Gy (*n* = 5), CpG ODN alone (*n* = 5), control GpC dinucleotides with 20 Gy (*n* = 5), or CpG ODN with 20 Gy (*n* = 5). Shannon entropy calculated from the abundance of TCR sequences captured by TCR sequencing and stratified by treatment group. Increasing entropy indicates reduced uniformity of TCR sequences. *P* values were calculated using 2-sided Wilcoxon tests. (**B**) TCR evenness is Shannon entropy normalized by species richness. (**C**) Immune-based classification of murine primary sarcomas. Sample sizes: muscle control (*n* = 3), tumor control (*n* = 5), CpG (*n* = 5), RT (*n* = 5), CpG+RT (*n* = 5).

Given that we observed activation and proliferation of CD8^+^ T cells, we next evaluated whether there was clonal T cell expansion. T cell receptor (TCR) clonality analysis was conducted on the bulk RNA-Seq data set and revealed that tumors treated with RT alone or CpG+RT have higher S-entropy scores, indicating increased infiltration of different clones of T cells into the tumor (Figure 4A). This is consistent with our findings of increased CD8^+^ T cells in tumors after combination therapy (Figures 2 and 3). TCR clonality assessment also demonstrated that sarcomas treated with combination treatment had a lower evenness score when compared with tumors receiving control treatment or CpG alone, possibly due to preferential tumor-antigen specific T cell expansion after treatment rather than pan–T cell proliferation (Figure 4B). It should be noted that CpG alone and RT alone also increase S-entropy and decrease evenness score for CD8^+^ T cells when compared with control. However, CpG+RT draws the most CD8^+^ T cells and, thus, attracts the highest number of tumor-antigen–specific T cells to the intratumoral area.

Petitprez and colleagues describe an immune-based classification (classes A–E) of STS based on the composition of the tumor microenvironment (43). Sarcoma immune classes (SIC) D and E are associated with improved survival, and SIC E is associated with a higher response rate to anti–PD-1 treatment. Using the bulk RNA-Seq, we assigned the murine p53/MCA tumors from each treatment group to an SIC based on the method described by Petitprez et al. (43) (Figure 4C). Mouse sarcomas treated with CpG and RT were all assigned to SIC D and E while sarcomas with no treatment were assigned to the less-inflamed SICs A, B, and C. Using CIBERSORTx and data from The Cancer Genome Atlas (TCGA), we compared the immune infiltration in human UPS samples from each SIC with murine p53/MCA tumors from each treatment group (Figure 4C). Similar to SIC D and E human tumors, mouse sarcomas treated with CpG and RT had the highest immune infiltration, further demonstrating the similarity between these tumors. It is interesting to note that even though mouse sarcomas treated with combination therapy closely resembled human SIC D and E tumors, in our experimental system, they did not respond to anti-PD1 treatment alone or in any combination with RT, CpG, and/or OX-40 agonist antibody (Supplemental Figure 6A). We included OX-40 because it was previously reported to act with CpG to stimulate the immune response in an autochthonous mouse model of breast cancer (44), but in our model system, OX40 was not active.

### Combination treatment of CpG and radiotherapy promotes myeloid cell remodeling and upregulates expression of MHC-I and MHC-II.

**Figure 5:**
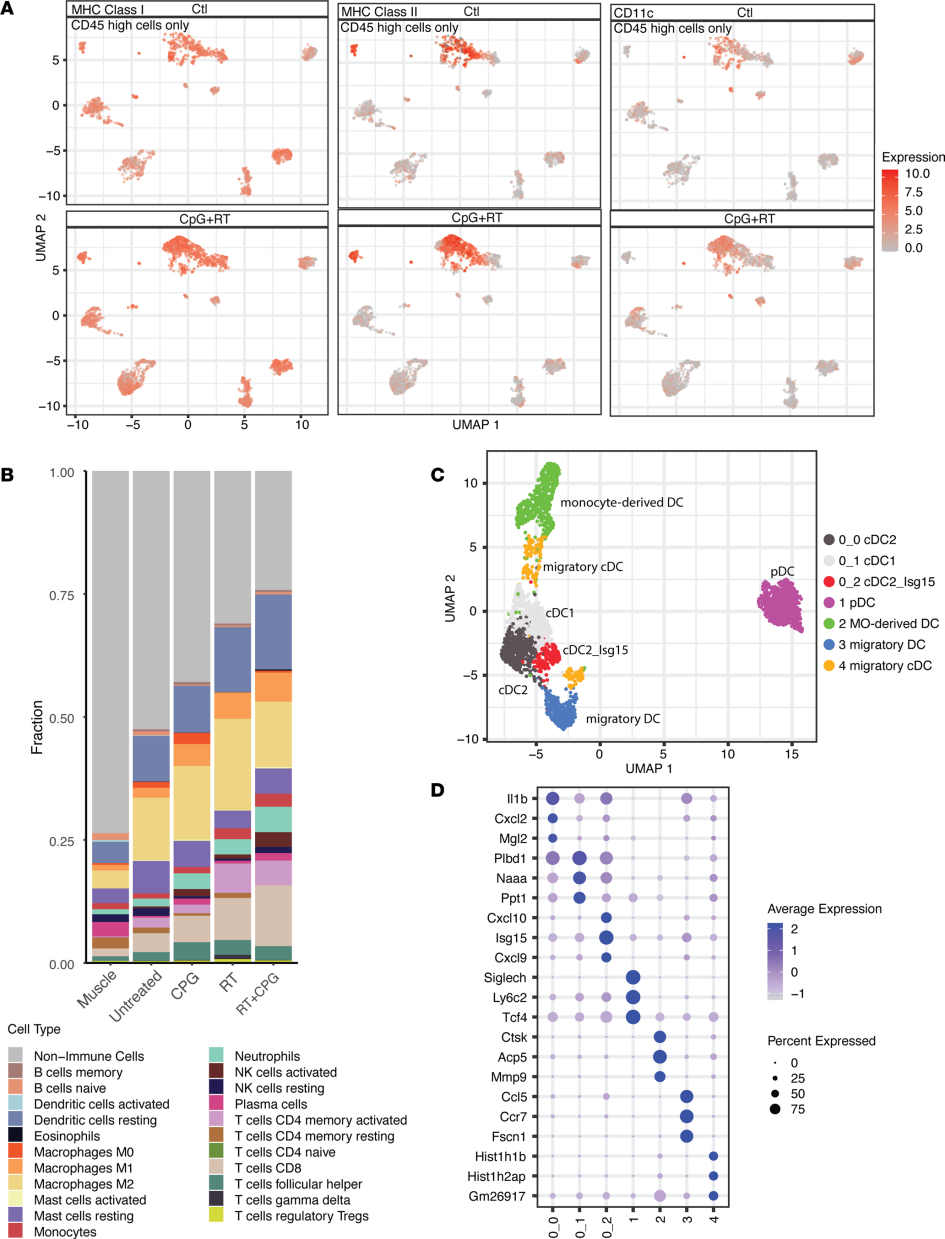
Treatment with CpG+RT promotes intratumoral myeloid cell remodeling. (**A**) UMAP plot of CyTOF clustering for CD45^hi^ cells from control (GpC dinucleotides) and CpG+RT treatment groups. MHC-I, MHC-II, and CD11c expression are highlighted in red. (**B**) Immune cell composition of all treatment groups from Bulk RNA-Seq. (**C**) UMAP plot of DC subclustering. (**D**) Bubble plot of top 3 differentially expressed genes in each of the DC subclusters. The shades of color are correlated with levels of expression. The sizes of circles are correlated with percentage of cells in that cluster that express the gene of interest. Sample sizes: tumor control (*n* = 5), CpG (*n* = 5), RT (*n* = 5), CpG+RT (*n* = 5).

We consistently observed an influx of CD8^+^ T cells in sarcomas treated with CpG and RT through CyTOF, scRNA-Seq, and bulk tumor RNA-Seq data. However, TLR9 is the canonical receptor for CpG, and it is constitutively expressed by B cells and plasmacytoid DCs rather than T cells. Therefore, we next used the CyTOF data to analyze TLR9-expressing antigen-presenting cells, as they could regulate the profound CD8^+^ T cell proliferation and trafficking into the tumor after CpG+RT. We observed that TLR9 protein expression is upregulated in RT alone and CpG+RT-treated tumors through CyTOF, especially in DCs and macrophages (Supplemental Figure 7A). We also observed upregulation of major histocompatibility complex (MHC) class I and class II proteins on antigen-presenting cells after treatment with CpG and RT through CyTOF (Figure 5A and Supplemental Figure 7A). A similar increase in intratumoral CD11c^+^ DCs was also observed with CpG+RT compared with control (Figure 5A and Supplemental Figure 7A). These results support the notion that an increase in DCs and proteins associated with antigen presentation pathways promotes the activation of CD8^+^ T cells after CpG+RT and, thus, enhances tumor killing. CIBERSORTx results also demonstrate expansions of M1 macrophages and activated DCs after combination treatment with CpG and RT (Figure 5B). It should be noted that RT seems to be the main driver for increased expression of TLR9, MHC-II, and CD11c. However, CpG+RT induced the most significant increase in MHC-I compared with control tumors, tumors treated with CpG alone, and tumors treated with RT alone; this corresponds with the most significant upregulation and activation of CD8^+^ T cells observed after CpG+RT combination therapy.

Unsupervised clustering analysis of DCs resulted in the identification of 7 subpopulations (Figure 5C), which we compared with publications for cell-type identification based on differential gene expression (Figure 5D, Supplemental Figure 8, and Supplemental Table 2). Cluster c0_0 is likely a group of proinflammatory conventional type 2 DCs (cDC2s) that express high *Itgam*, *Il1b*, *Cd14*, and *Tnf*, as previously identified by Cheng and colleagues (45). Cluster c0_1 closely resembles cDC1s with differential expression of *Itgae*, *Xcr1*, and *Clec9a* (45). CD103^+^ cDC1s have been shown to transport intact antigens to the tumor-draining lymph nodes and activate CD8^+^ T cells. Cluster c0_2 is characterized by increased expression of chemokine and IFN-inducible genes, such as *Cxcl10*, *Ifit1*, and *Isg15*, which were previously identified as cDC2_Isg15 (45).

Cluster c1 clustered farther apart from all other DCs and expresses signature genes representing plasmacytoid DCs (pDC), such as *Siglech*, *Ly6c2*, *Bst2*, *Ptprcap*, and *Tcf4* (Figure 5D and Supplemental Figure 8) (46, 47). C3 resembles migratory DCs with high expression of *Ccl5*, *Ccr7*, *Ccl22*, and *Cacnb3* (48–50). None of these populations had substantial changes in size based on treatment groups (Supplemental Figure 7B).

Cluster c2 is likely a population of monocyte-derived DCs that expresses *Ctsk*, which plays important roles in regulating DC maturation, enhancing DC–T cell interactions, and promoting TLR9-induced cytokine production (51–53). It is worth noting that c2 almost completely disappears after treatment with RT or CpG+RT (Supplemental Figure 7B). Since both bulk tumor RNA-Seq and CyTOF data demonstrate increased infiltration of DCs after combination treatment, it is plausible to hypothesize that c2 migrated to the lymph nodes for antigen presentation after CpG+RT. However, it is also possible that c2 was sensitive to radiation and was eliminated after treatment. Cluster c4 is another DC population that decreased after combination treatment (Figure 5D and Supplemental Figure 7B), and it expresses *Itgam* (CD11b) but not *Itgae* (CD103), which is the phenotype often associated with migratory cDCs that travel to lymph nodes for antigen presentation (54, 55).

To begin to investigate the role of myeloid cell populations in mediating treatment effects, we used the scRNA-Seq data to perform cell-cell communication analyses between myeloid cell populations and T cells (Supplemental Figure 9, A and B). Several intercell communication pathways changed after CpG+RT treatment. There is a high communication probability between THBS1–CD47 monocyte–T cells pair in the untreated tumors. After treatment with CpG+RT, there is less interaction between THBS1–CD47 (Supplemental Figure 9A). THBS1 is often found to be associated with tumor progression, metastasis, and therapy resistance in many different types of cancers (56–59). A recent study shows that monocytes are the primary source of THBS1 in colorectal cancer and that they contribute to the development of metastasis by inducing cytotoxic T cell exhaustion (56). After combination treatment with CpG and radiotherapy, new interactions between H2-k1–CD8a, H2-d1–CD8a, and CXCL4/9/10/16–CXCR3/6 were observed (Supplemental Figure 9A). Robust communications between H2-k1–CD8a/b1 and H2-d1–CD8a/b1 represent increased intratumoral antigen presentation by myeloid cell populations to T cells (60–62). Increased interactions between CXCL4/9/10/16–CXCR3/6 may promote the recruitment of T cells to the intratumoral area (19, 21, 63–67). These findings are consistent with a model where CpG+RT treatment promoted DC maturation by upregulating genes that are associated with antigen presentation, such as MHC-I and MHC-II. In summary, CellChat analysis indicates that the combination treatment induced upregulation of genes and cellular communications associated with antigen presentation and T cell trafficking.

### Lymphocytes, especially CD8^+^ T cells, are crucial in mediating the antitumor effects of CpG and radiotherapy in vivo.

**Figure 6:**
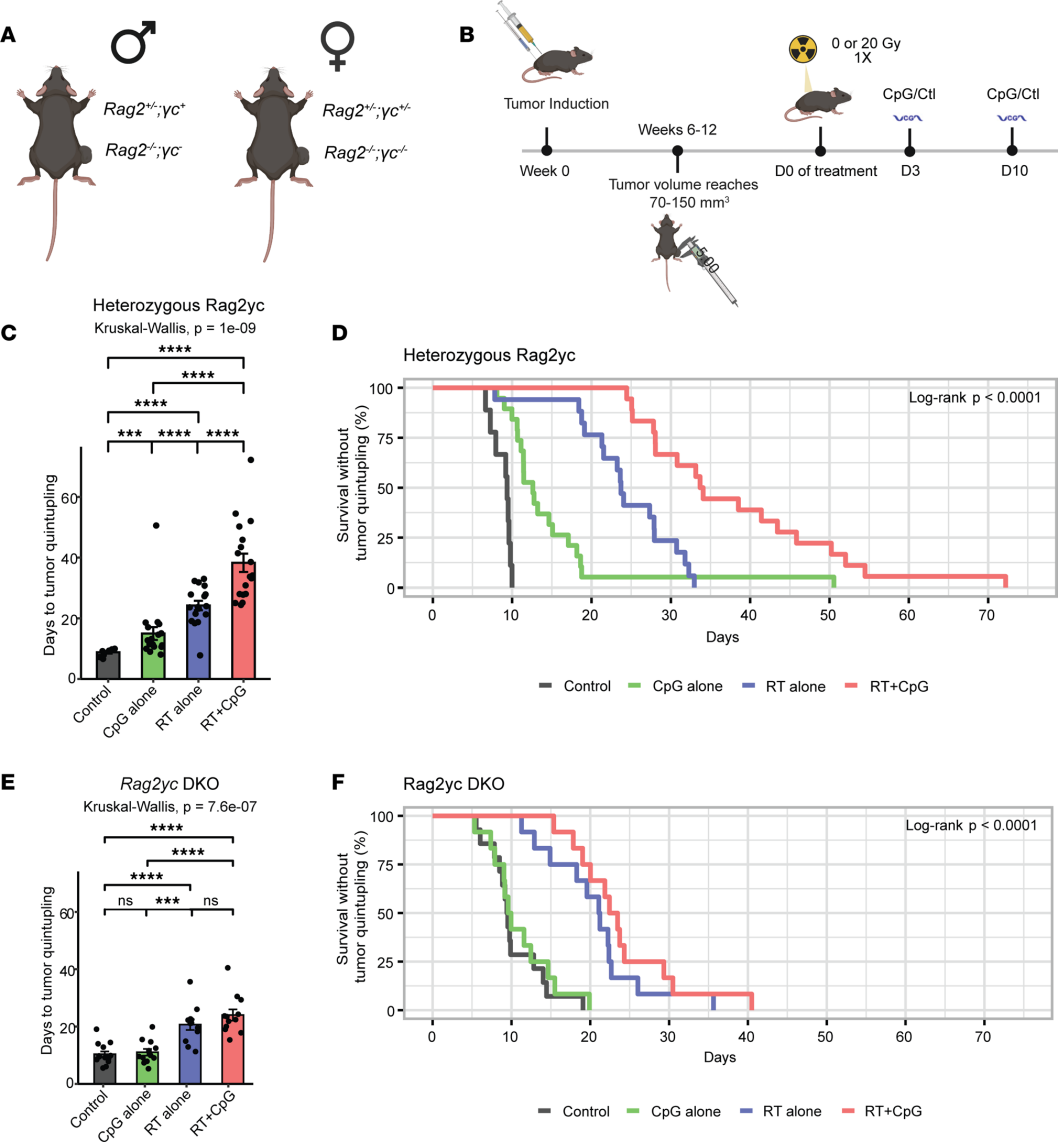
Lymphocytes mediate the antitumor effects of the combination treatment CpG+RT. (**A**) Primary sarcoma initiation by intramuscular injection of Adeno-Cas9-sgp53 and MCA. (**B**) Autochthonous sarcoma develops at the injection site about 7–11 weeks after induction. Mice were treated with CpG ODN or control GpC dinucleotides and 0 or 20 Gy when tumors reached > 70 mm^3^. (**C**) Heterozygous mice (*Rag2^+/–^*;*yc^+^* or *Rag2^+/–^*;*yc^+/–^*) with p53/MCA sarcomas received control GpC dinucleotides with 0 Gy (black, *n* = 9), CpG ODN alone (green, *n* = 17), control GpC dinucleotides with 20 Gy (blue, *n* = 17), or CpG ODN with 20 Gy (red, *n* = 18). Figure shows time to tumor quintupling (days). (**D**) Heterozygous mice (*Rag2^+/–^*;*yc^+^* or *Rag2^+/–^*;*yc^+/–^*) with p53/MCA sarcomas received control GpC dinucleotides with 0 Gy (black, *n* = 9), CpG ODN alone (green, *n* = 17), control GpC dinucleotides with 20 Gy (blue, *n* = 17), or CpG ODN with 20 Gy (red, *n* = 18). Figure shows time to tumor quintupling (days). (**E**) Homozygous mice (*Rag2^–/–^*;*yc^–^* or *Rag2^–/–^;yc^–/–^*) with p53/MCA sarcomas received GpC dinucleotides control with 0 Gy (black, *n* = 14), CpG ODN alone (green, *n* = 12), GpC dinucleotides control with 20 Gy (blue, *n* = 12), or CpG ODN with 20 Gy (red, *n* = 12). Figure shows time to tumor quintupling (days). (**F**) Homozygous mice (*Rag2^–/–^*;*yc^–^* or *Rag2^–/–^*;*yc^–/–^*) with p53/MCA sarcomas received GpC dinucleotides control with 0 Gy (black, *n* = 14), CpG ODN alone (green, *n* = 12), GpC dinucleotides control with 20 Gy (blue, *n* = 12), or CpG ODN with 20 Gy (red, *n* = 12). Figure shows time to tumor quintupling (days). Kruskal-Wallis test was used for the group comparison, while the Wilcoxon test was selected for the pair-wise comparisons. ***P ≤ 0.001, ****P ≤ 0.0001.

To evaluate whether the CpG+RT treatment effect is indeed mediated through the adaptive immune system in vivo, we induced p53/MCA tumors in *Rag2^–/–^*;γ*c^–^* (male) or *Rag2^–/–^*;γ*c*^–/–^ (female) and their littermate controls *Rag2^+/–^*;γ*c^+^* (male) or *Rag2^+/–^*;γ*c^+/–^* (female) (Figure 6A). Since the γ*c* gene is X-linked, the genotypes for littermate controls are different between males and females. *Rag2^–/–^*;γ*c^–^* (male) and *Rag2^–/–^*;γ*c*^–/–^ (female) mice are incapable of generating functional B cells, T cells, or NK cells. When tumor volume reached 70–150 mm^3^, mice were randomized to receive 0 Gy or 20 Gy RT (D0) and CpG or control (GpC dinucleotides with the positions of cytosine and guanine reversed relative to the phosphate linker) (D3 and D10) (Figure 6B). The increase in time to tumor quintupling with CpG+RT compared with control was similar for heterozygous littermate controls that retained functional B cells, T cells, or NK cells (Figure 6, C and D) and 129/SvJae mice (Figure 1C). However, this treatment effect of CpG+RT was lost in homozygous *Rag2*γ*c*-double KO (*Rag2*γ*c*-DKO) mice (*Rag2^–/–^*;γ*c^–^* [male] and *Rag2^–/–^*;γ*c*^–/–^ [female]) (Figure 6, E and F), suggesting that the adaptive immune system plays a crucial role in facilitating the antitumor effects of adding CpG to RT.

**Figure 7:**
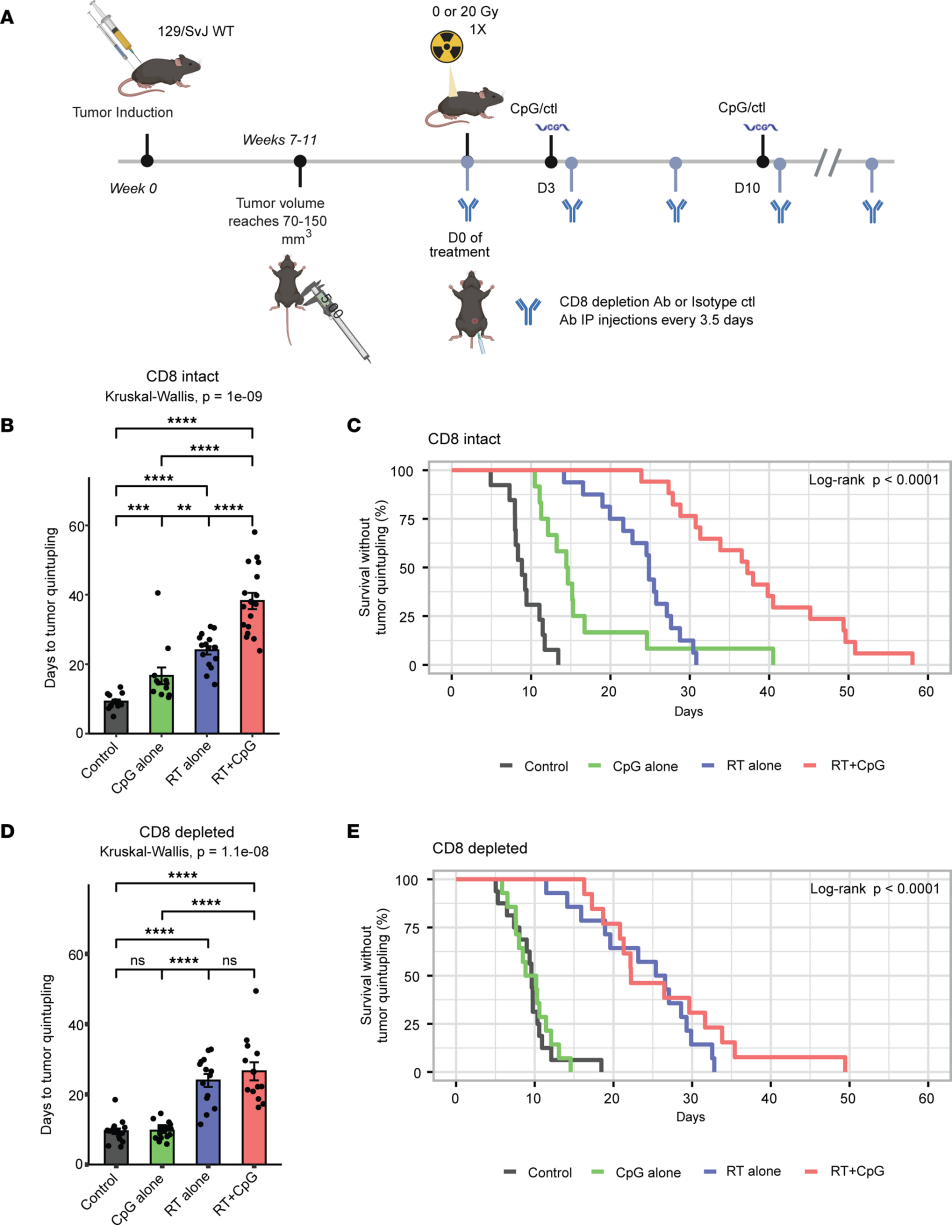
CD8 T cells are required for the treatment effects of CpG+RT. (**A**) Primary sarcoma initiation by intramuscular injection of Adeno-Cas9-sgp53 and MCA. Autochthonous sarcoma develops at the injection site about 7–11 weeks after induction. Mice were treated with CpG ODN or control GpC dinucleotides and 0 or 20 Gy when tumors reached > 70 mm^3^. Mice received i.p. CD8 isotype control or CD8 depletion Ab on the same day tumors received RT. CD8 isotype control or CD8 depletion Ab are repeated every 3.5 days until tumor size reached humane endpoint. (**B**) 129/SvJ mice with p53/MCA sarcomas, injected with CD8 isotype control Ab, received GpC dinucleotides control with 0 Gy (black, *n* = 13), CpG ODN alone (green, *n* = 12), GpC dinucleotides control with 20 Gy (blue, *n* = 15), or CpG ODN with 20 Gy (red, *n* = 14). (**C**) 129/SvJ mice with p53/MCA sarcomas, injected with CD8 isotype control Ab, received GpC dinucleotides control with 0 Gy (black, *n* = 13), CpG ODN alone (green, *n* = 12), GpC dinucleotides control with 20 Gy (blue, *n* = 15), or CpG ODN with 20 Gy (red, *n* = 14). (**D**) 129/SvJ mice with p53/MCA sarcomas, injected with CD8 depleting Ab, received GpC dinucleotides control with 0 Gy (black, *n* = 16), CpG ODN alone (green, *n* = 14), GpC dinucleotides control with 20 Gy (blue, *n* = 13), or CpG ODN with 20 Gy (red, *n* = 11). (**E**) 129/SvJ mice with p53/MCA sarcomas, injected with CD8 depletion Ab, received GpC dinucleotides control with 0 Gy (black, *n* = 16), CpG ODN alone (green, *n* = 14), GpC dinucleotides control with 20 Gy (blue, *n* = 13), or CpG ODN with 20 Gy (red, *n* = 11). Figure shows time to tumor quintupling (days). Kruskal-Wallis test was used for the group comparison, while the Wilcoxon test was selected for the pair-wise comparisons. **P ≤ 0.01, ***P ≤ 0.001, ****P ≤ 0.0001.

We next directly tested if CD8^+^ T cells are necessary for the treatment effects observed with CpG+RT through CD8^+^ T cell depletion in 129/SvJae mice with p53/MCA sarcomas. When tumors reached 70–150 mm^3^, mice received i.p. injections of isotype control or anti-CD8 antibodies on the same day as RT or sham RT (Figure 7A). Isotype control or CD8-depleting antibodies are repeated every 3–4 days until euthanasia upon reaching the humane endpoint (Figure 7B). Tumor growth delay was observed with CpG+RT in the isotype control group (Figure 7, B and C), but the growth delay with combination treatment was not observed in the CD8-depleted mice (Figure 7, D and E). These results demonstrate the essential role of CD8^+^ T cells in tumor growth delay induced by CpG+RT in combination. Although TLR9 is the canonical receptor for CpG, it is possible that the effects of CpG are mediated through a different mechanism. Therefore, to investigate the role of TLR9 receptors in mediating the treatment effects of the combination of CpG+RT, we induced p53/MCA tumors in WT *TLR9^+/+^* littermate controls and mice lacking 1 (*TLR9^+/–^* mice) or both (*TLR9^–/–^* mice) alleles for the TLR9 receptor (Supplemental Figure 10A). When tumor volume reached 70–150 mm^3^, mice were randomized to receive 20 Gy RT (D0) and CpG or control on D3 and D10 (Supplemental Figure 10A). TLR9^–/–^ mice lost their sensitivity to the combination of CpG+RT (Supplemental Figure 10B). Interestingly, the response of p53/MCA sarcomas to CpG+RT was also diminished in TLR9^+/–^ mice (Supplemental Figure 10C), suggesting that the level of TLR9 receptor plays a critical role in mediating the treatment response to CpG+RT (Supplemental Figure 10D).

## Discussion

Many studies have explored synergistic effects between immunotherapies and RT in sarcoma (3, 8, 9). However, most preclinical experiments are conducted with xenograft or transplanted models in which the tumor does not coevolve with the host immune system. Therapeutic approaches that elicit impressive survival benefits in transplanted tumor models may fail when translated into clinical trials (12, 68, 69). In this study, we addressed the limitations of transplant tumor models by utilizing the high mutational load autochthonous p53/MCA murine sarcoma model, which allows the tumor to develop under the surveillance of an intact immune system (10, 11). p53/MCA sarcomas mimic the histology of human UPS, which is one of the more responsive sarcoma subtypes to immunotherapy treatments (4, 10, 13).However, the chemically induced p53/MCA sarcomas have a much greater number of nonsynonymous somatic mutations when compared with most human UPS (11, 70, 71). We have previously performed bulk tumor RNA-Seq on primary p53/MCA sarcomas and transplant p53/MCA sarcomas (12). CIBERSORTx analysis of the gene expression data from the 2 different murine tumor models was compared with human UPS samples from TCGA, and primary p53/MCA tumors resembled the less-inflamed sarcomas in patients with low immune infiltration and had a worse correlation with overall survival (12, 43). In contrast, untreated transplant p53/MCA tumors resembled highly inflamed human sarcomas with high levels of immune infiltrate and are associated with improved overall survival (12, 43).

Our results demonstrate an enhanced radiation response of primary sarcomas treated with intratumoral CpG as measured by tumor growth delay, which is mediated through the activation and expansion of intratumoral CD8^+^ T cells. Our work suggests that the combination of RT with a TLR9 agonist, such as CpG, warrants evaluation in a clinical trial of sarcomas and perhaps other cancers. Mechanistically, our work indicates that combination treatment with CpG and RT enhances the activation and proliferation of intratumoral CD8^+^ T cells, as demonstrated by CyTOF, IHC, scRNA-Seq, and bulk tumor RNA-Seq. These CD8^+^ T cells express high levels of granzymes and IFN-γ, indicating that they are activated and capable of cellular cytotoxicity. Furthermore, the majority of these T cells are also in the S or G2-M phases of the cell cycle, demonstrating active proliferation. The TCR clonality analysis further supports targeted tumor-antigen–specific T cell response rather than a general proliferation of T cells. Additionally, our findings show that p53/MCA sarcomas in mice treated with CpG and RT exhibit immune profiles similar to SIC classes D and E in patients with UPS and those classes are associated with better survival outcomes and response rates to anti-PD1 therapy in patients with STS. Furthermore, CD8^+^ T cell depletion in the murine model abrogated the treatment effect of CpG+RT, which establishes a critical role of CD8^+^ T cells in mediating the treatment effects of this combination therapy.

It should be noted that RT seems to be the main driver for the differential expression of TLR9, MHC-II, and CD11c in myeloid cells, while CpG is the main driver for Granzyme B expression in CD8 T cells. Although the expression of specific genes after combination CpG and RT treatment does not always differ significantly from tumors treated with CpG alone or RT alone, it is the combination of changes after RT, including increased TLR9 expression with the TLR9 agonist CpG, that is necessary for maximal CD8 T cell infiltration, which is required for maximal tumor response. Future functional studies are needed to determine the underlying mechanisms responsible for CD8^+^ T cell activation and trafficking to the tumor after CpG and RT that is distinct from CpG alone or RT alone. Sagiv-Barfi and colleagues explored the combined therapeutic effects of the administration of local TLR9 agonist with systemic anti-OX40 agonist in murine models with spontaneous mammary gland tumors (44). They observed significant tumor burden reduction not only at the TLR9 agonist injection site but also at distant tumor sites. They reported upregulated OX40 expression on CD4^+^ T cells after CpG treatment and superior treatment response when anti-OX40 was added to intratumoral CpG injections (44). However, we did not observe increased OX40 expression after CpG+RT (Supplemental Figure 6B), nor did we observe a synergistic treatment effect with anti-OX40 in the p53/MCA sarcoma model (Supplemental Figure 6A). Seo and colleagues recently published results from a phase I clinical trial utilizing intratumoral injection of a TLR4 agonist and radiotherapy to treat 12 patients with metastatic sarcoma (72). They observed significantly elevated intratumoral infiltration of CD4^+^ T cells and tumor-antigen–specific clonal expansion of this population (72). In contrast, in the p53/MCA model, we did not observe substantial changes in the intratumoral CD4^+^ T cell population after combination of CpG+RT, suggesting potential differences in immune-activation mechanisms between TLR4 and TLR9 pathways (Figure 3E and Figure 5B) and/or differences in the mouse model and human sarcomas.

There were some differences in the cell population changes after CpG+RT treatment in scRNA-Seq data versus bulk tumor RNA-Seq and those differences might stem from different rates of mRNA recovery in the sample preparation process. For example, both bulk tumor RNA-Seq and CyTOF data demonstrate an increase in DC populations after combination treatment with CpG+RT. However, scRNA-Seq generally showed a decrease in the number of infiltrating DCs after combination treatment, suggesting a technical limitation for detecting DCs with scRNA-Seq in our experiments. 10X Genomics reported that about 30%–32% of mRNA transcripts are captured per cell utilizing the Single Cell 3′ reagent chemistry v3 (73). However, Qiagen reports more than 90% mRNA recovery from tissues utilizing their mRNA extraction kit (74). Therefore, differences in mRNA recovery could partly account for discrepancies in DC populations observed between bulk tumor RNA-Seq and scRNA-Seq data.

Overall, CpG appears to be an excellent candidate for the treatment of patients with STS due to its ease of local administration and favorable safety profile (75, 76). No apparent toxicity was observed in mice treated with CpG+RT. Furthermore, our in vivo studies with p53/MCA sarcomas demonstrated that using TLR9 agonists in conjunction with radiotherapy significantly outperformed the individual treatments or no treatment in terms of delaying tumor progression. As previously mentioned, Seo and colleagues recently demonstrated that metastatic sarcoma lesions treated with the combination therapy of TLR4 agonist and radiotherapy achieved durable local control (72). Similarly, our in vivo studies in mice with p53/MCA sarcomas demonstrated that intratumoral injection of CpG as a TLR9 agonist in conjunction with radiotherapy significantly improved response compared with either treatment alone as measured by tumor growth delay. Our results with CpG+RT and the initial clinical trial of a TLR4 agonist with RT demonstrate the potential effectiveness of TLR agonists and radiotherapy in treating sarcomas. Furthermore, unlike other immunotherapies, such as immune checkpoint inhibitors that aim to reverse the exhaustion state of T cells, TLR9 agonist combined with RT causes activated CD8^+^ T cells that are not yet exhausted to infiltrate the tumor and enhance the radiation response. This study supports translating the therapeutic approach of radiotherapy with CpG or another TLR9 agonist into clinical trials for patients with STS.

## Methods

### Sex as a biological variable

These studies included both male and female animals.

### Experimental models details

The *Rag2^–/–^;*γc*^–/–^* mice, *TLR9^–/–^* mice, and their littermates were purchased from The Jackson Laboratory and bred at Duke University with a mixed BALB/cAnNTac and 129S4/SvJae background. WT 129S4/SvJae mice used in this study were also purchased from The Jackson Laboratory and bred at Duke University. To minimize the effects of sex and genetic background, male and female mice and age-matched littermate controls were used for every experiment so that potential genetic modifiers would be randomly distributed between experimental and control groups.

### Sarcoma induction and treatment

Primary p53/MCA sarcomas were generated in 129S4/SvJae, *Rag2^–/–^;*γc*^–/–^*, *TLR9^–/–^,* and littermate control mice between 6 and 12 weeks old by intramuscular injection of adenovirus expressing Cas9 and sgRNA targeting *Trp53* (Adeno-p53-sgRNA; Viraquest) into mice as previously described (12). Ddenovirus (25 μL) was mixed with 600 μL DMEM (Thermo Fisher Scientific) and 3 μL 2M CaCl_2_, and it was then incubated for 15 minutes at room temperature prior to injection. The prepared mixture (50 μL) was injected into the gastrocnemius muscle, followed by injection of 300 μg MCA (MilliporeSigma) resuspended in sesame oil (MilliporeSigma) at 6 μg/μL.

For tumor growth delay studies in 129S4/SvJae, *Rag2^–/–^;*γc*^–/–^*,*TLR9^–/–^*, and littermate control mice, the mice were randomized to treatment groups when tumors reached 70–150 mm^3^ (D0). Tumors were monitored 3 times weekly by caliper measurements in 2 dimensions until 1 dimension of the tumor reached 15 mm. Mice received 1 dose of 0 or 20 Gy of image-guided RT to the tumor-bearing hind limb on D0. Mice were anesthetized with 2% isoflurane and 98% oxygen at 2 L/min and held on the specimen positioning stage of a μCT on a Small Animal Radiation Research Platform. The first half of tumor quintupling studies and scRNA-Seq and bulk tumor RNA-Seq experiments were conducted utilizing SmART+, Precision Inc. The second half of tumor quintupling studies and CyTOF experiments were conducted using SARRP, Xstrahl Inc. The right hind limb was identified using μCT guided fluoroscopy (60 kVp, 0.8 mA x-rays using a 1 mm Al filter). Irradiations were performed using parallel-opposed anterior and posterior x-rays, which were delivered via 20 mm × 20 mm collimators (220 kVp, 13 mA x-rays using a 0.15 mm Cu filter) with a dose of 20 Gy of radiation prescribed to mid-plane delivered in a single, unfractionated dose.

In total, 50 μL of CpG or GpC control was injected intra- and peritumorally on D3 and D10. CpG (InvivoGen, ODN 1826) or GpC control with the positions of cytosine and guanine reversed relative to the phosphate linker (InvivoGen, ODN 2138) was diluted in endotoxin-free water at 1 mg/mL. Antibodies were administered starting on D0 (on the same day as RT) by i.p. injection of 200 μL per dose at 1 mg/mL diluted in PBS. Anti-CD8 (BioXCell, BE0061) or isotype control (BioXCell, BE0090) antibodies were injected every 3–4 days for the duration of the experiment. Anti–PD-1 (BioXCell, BE0146) and isotype control (BioXCell, BE0089) were injected on D3, D7, and D10. Anti-OX40 (Bristol Myers Squibb) or isotype control (Bristol Myers Squibb) were injected on D3 and D10.

### Mass cytometry

#### Tumor harvest and dissociation.

Tumors were dissected from mice, minced, and digested using the Miltenyi Biotec tumor dissociation kit (mouse, tough tumor dissociation protocol) for 40 minutes at 37°C. Cells were then strained through a 70 μm filter and washed with Maxpar Cell Staining Buffer (CSB) (Standard Bio Tools). RBCs were lysed using ACK lysis buffer (Lonza). Cells were then washed and resuspended in Maxpar PBS for cell counting using Trypan Blue (Thermo Fisher Scientific).

#### CyTOF Staining.

For custom-conjugated antibodies, 100 μg of antibody was coupled to Maxpar X8 metal-labeled polymer according to the manufacturer’s protocol (Standard Bio Tools). After conjugation, the metal-labeled antibodies were diluted in Antibody Stabilizer PBS (Candor Bioscience) for long-term storage according to the manufacturer’s protocol. After tumor dissociation and RBC lysis as described above, 3 million cells per sample were transferred to 5 mL round-bottom tubes (Corning). Cells were incubated with 300 μL of Cell-ID Cisplatin-195Pt (Standard Bio Tools) diluted 1:8,000 in Maxpar PBS (Standard Bio Tools) for 5 minutes at room temperature before being washed with CSB (Standard Bio Tools). Samples were incubated with 1.5 μg TruStain FcX PLUS Blocking Reagent (BioLegend) for 10 minutes at room temperature; then, 56.55 μL extracellular antibody cocktail was added and incubated for 30 minutes at room temperature. The final staining volume was 130 μL, including residual CSB from the wash, FcX PLUS blocking reagent, CSB, and antibody cocktail. Cells were washed twice with CSB and were then fixed and permeabilized with Foxp3/Transcription Factor Fixation/Permeabilization Buffer (eBioscience) overnight at 4°C. The next morning, cells were washed twice with permeabilization buffer (eBioscience). In total, 50 μL of intracellular antibody cocktail in permeabilization buffer was added and incubated for 30 minutes at room temperature, followed by 2 washes with permeabilization buffer. Cells were fixed in 1.6% methanol-free paraformaldehyde (PFA) (Thermo Fisher Scientific) diluted with Maxpar PBS (Standard Bio Tools) for 10 minutes at room temperature. Samples were incubated for 1 hour in Maxpar Fix and Perm Buffer (Standard Bio Tools) with 1 mL of 157.2 nM Cell-ID Intercalator (Standard Bio Tools) containing 191Ir and 193Ir. After staining, samples were centrifuged (at 800 g for 5 minutes) and resuspended in 100 μL of residual Intercalator/Fix and Perm Buffer. Cells were transferred to 1.5 mL microcentrifuge tubes and stored at –80°C. On the day of acquisition, cells were washed once with CSB and once with Cell Acquisition Solution (CAS) (Standard Bio Tools) before being filtered and diluted in CAS containing 10% EQ Calibration Beads (Standard Bio Tools) at 0.5 million cells per mL before acquisition on a mass cytometer (Helios).

#### CyTOF data analysis.

Mass cytometry data were analyzed using Standard Bio Tools CyTOF software (v7.0). Individual samples were gated in Cytobank to exclude beads, debris, dead cells, and doublets for further analysis. For each experimental group (treatment), cells from 8 to 11 tumors per group were manually gated to identify specific populations.

#### CyTOF dimension reduction and clustering.

Expression data from all .fcs files were log_2_ transformed. We used unbiased clustering with FastPG v0.0.8 to identify clusters of cells in the expression data (77). A k of 50 was used, and all cells were included (CD45^hi^ and CD45^lo^) in the clustering step. Dimension reduction was performed on the log_2_ expression data using both PCA and UMAP approaches. The statistical significance of markers across treatment groups was determined using a Wilcoxon rank-sum test.

### Bulk tumor RNA-Seq of p53/MCA sarcomas in 129S4/SvJae mice

#### RNA extractions.

Tumor specimens and matched muscle control were harvested and stored in RNALater (Ambion) at −80°C until all samples were collected. RNA extractions from each sample were performed using RNeasy Fibrous Tissue Mini Kit (Qiagen). Extracted total RNA quality and concentration were assessed on a NanoDrop Spectrophomoter (Thermo Fisher Scientific). RNA-Seq libraries were prepared using TruSeq Small RNA Library Preparation Kits (Illumina) following the manufacturer’s protocol. RNA-Seq was performed on an Illumina Novoseq in 151 base pair, paired-end configuration. Greater than 50,000 reads per cell were collected per the manufacturer’s recommendation.

#### Sequence alignment and SIC labels.

All FASTQ files were processed using a combination of STAR v2.7.10a and Salmon v1.2.0 and were aligned with mouse build GRCh38 - mm10, release 84, as a reference. GENCODE vM10 was used for transcriptome annotation (78, 79). FASTQ files from the same sample but different lanes were merged. Reads were mapped using STAR v2.7.10a. Transcripts were counted with Salmon v1.2.0 and Picard v 2.27.4 was utilized for quality control (80). Mouse genes were mapped to their corresponding human orthologs using the ‘biomaRt’ R package (81). The expression matrix was restricted to protein-coding genes, and expression levels per gene were summarized as transcripts per million (TPM). Preprocessed bulk RNA-Seq profiles from human UPS from TCGA were downloaded and scaled to TPM. SIC labels were obtained from the authors (43, 71).

#### CIBERSORTx RNA-Seq analysis.

Cell type proportions were inferred using CIBERSORTx with 2 previously published signature matrices (LM22 and TR4) (42). LM22 encompasses 22 distinct human immune cell subsets, and TR4 comprises epithelial, fibroblast, endothelial, and immune cells (42, 82). Bar plots and heatmaps were visualized with the ggplot2 and ComplexHeatmap R packages, respectively (83). For the heatmaps, abundances were normalized to mean zero and unit variance. *P* values were calculated using 2-sided Wilcoxon tests.

#### Assignment of mouse sarcomas to SIC.

SIC were assigned to normal muscle and mouse sarcomas in each treatment group as described previously (43). Briefly, abundance scores for T cells, CD8 T cells, cytotoxic lymphocytes, B cell lineage, NK cells, monocytic lineage, myeloid DCs, neutrophils, and endothelial cells were determined from the bulk RNA-Seq using MCP-counter (84) and normalized across samples. Because the transcriptional profiles of the mouse tumors in this study are most similar to human UPS, we constructed centroids using the MCP-counter *Z* scores for UPS from TCGA (71) based on the SIC labels obtained from the authors (43). Mouse samples were assigned to the closest SIC by evaluating the Euclidean distance to each centroid.

#### TCR reconstruction and CDR3 metrics.

TRUST4 v1.0.12 (Tcr Repertoire Utilities for Solid Tissue) were used to reconstruct TCR and BCR sequences from BAM files generated from the STAR/Salmon outputs (85). All parameters for TRUST4 were set to default. The final output from TRUST4 was summary matrices for each sample that included hypervariable complementarity-determining region 3 (CDR3) nucleotide and amino acid sequences, counts, frequencies, and V, D, and J chain names. Using the outputs from TRUST4, we calculated the following diversity metrics for all CDR3 species per treatment group: Shannon entropy and evenness by sequence were used to compare TCR species across treatment groups (86, 87). Shannon entropy measures the richness and abundance of each species. Species evenness measures the uniformity of the species. The calculations for each metric given a single TCR species are as follows:

Count proportion: species count / total species count

Species richness: nucleotide length

Shannon entropy: –Σ (count proportion × log[count proportion])

Evenness by sequence: Shannon entropy / log(species richness)

scRNA-Seq of p53/MCA sarcomas in 129S4/SvJae mice

#### Tumor harvest and dissociation.

Tumors were dissected from mice, minced, and digested using the Miltenyi Biotec tumor dissociation kit (mouse, tough tumor dissociation protocol) for 40 minutes at 37°C. Cells were then strained through a 70 μm filter and washed with FACS buffer (HBSS [Thermo Fisher Scientific] with 5 mM EDTA [Sigma-Aldrich] and 2.5% FBS [Thermo Fisher Scientific]). RBCs were lysed using ACK lysis buffer (Lonza) and washed again with FACS buffer.

#### FACS.

Dissociated cells were prepared for FACS of CD45^+^ cells for scRNA-Seq. Single-cell suspensions of tumor tissues were blocked with 1.5 μg TruStain FcX PLUS Blocking Reagent (BioLegend) for 10 minutes at room temperature before being stained with Live/Dead dye (Zombie Aqua, BioLegend) and anti–mouse CD45 (APC-Cy7, BioLegend) for 25 minutes on ice. Live CD45^+^ cells were isolated for scRNA-Seq using an Astrios (Beckman Coulter) sorter and resuspended in PBS with 0.04% BSA at a concentration of 1,000 cells/μL for scRNA-Seq.

#### Library preparation and sequencing.

Single-cell suspensions from sorted live CD45^+^ cells were loaded on a Chromium Controller (10X Genomics) to generate single-cell beads in emulsion, and scRNA-Seq libraries were prepared using the Chromium Single Cell 3′ Reagent Kits (v3.1 Single Index Kit), Chromium Next GEM Single Cell 3′ GEM, Library & Gel Bead Kit v3.1, Chromium Next GEM Chip G Single Cell Kit, and Single Index Kit T Set A (10X Genomics) following the manufacturer’s protocol. Single-cell barcoded cDNA libraries were qualified and quantified using Agilent Bioanalyzer. cDNA libraries were sequenced on an Illumina NextSeq 500. Read lengths were 26 bp for read 1, 8 bp for i7 index, and 98 bp for read 2. Ten thousand cells from each sample were sequenced with greater than 50,000 reads per cell as recommended by the manufacturer.

#### Analysis of scRNA-Seq data.

Raw FASTQ files were mapped to *Mus musculus* reference mm10 GRCm38 using CellRanger v6.1.1 (10X Genomics) with default parameters and GENCODE vM23 for transcriptome annotation. Expression matrix assembly and calculation of cell metrics (gene counts, molecule counts, percent mitochondrial genes) were performed using Seurat v.4.1.0 (88). We removed low-quality cells that had total nonzero gene counts ≤ 500 and ≥ 5,000; total nonzero molecule counts ≤ 2,000 and ≥ 40,000; ≥ 10% mitochondrial gene presence; a hybrid doublet score ≥ 1.0 per SCDS default parameters (89); and an ambient RNA contamination score ≥ 0.50 per decontamX default parameters (90). Count data were normalized using LogNormalize in Seurat with a scaling factor of 10,000. The top 10,000 variable features were then used to scale the data using Seurat’s ScaleData function. Although cell cycle genes were scored using CellCycleScoring in Seurat, no variables were regressed out upon scaling. Cell types were annotated using SingleR v.1.8.1 and the mouse immunological genome project data set (Immgen) available in the celldex v.1.4.0 library as a reference (91). Dimension reduction was performed using Harmony v0.1.0 with RunHarmony set to theta of 2 and all other parameters set to default (92). Harmony reductions were used for all downstream UMAP and clustering calculations. To select a clustering resolution, we calculated the adjusted rand index (ARI) and selected the clustering resolution with the highest ARI. Additional subclustering was performed downstream on cell types individually, and T cell subtypes were annotated using the T cell atlas from projecTILs with filter.cells set to “TRUE” (93). DEGs were identified using the FindAllMarkers in Seurat with only.pos set to TRUE, logfc.threshold set to 0.1, and min.pct set to 0.25.

### IHC staining

Parts of the sarcoma were preserved for IHC staining when tumors were harvested for bulk tumor RNA-Seq. Small chunks of sarcoma were fixed in 10% formaldehyde overnight and then preserved in 70% ethanol until paraffin embedding. Formalin-fixed, paraffin-embedded tissues were sectioned and fixed onto slides for staining. Tissue sections were then deparaffinated by xylene and rehydrated with a series of graded ethanol and tap water. Slides were cooked in a rice cooker with Antigen Unmasking Solution, Citric Acid Based (Vector Laboratories) for epitope retrieval. After washes with tap water and PBS, tumor sections were incubated in normal goat serum for 30 minutes at room temperature and then with CD8 antibody (Cell Signaling Technology, 98941) at 4°C overnight. The following day, slides were washed with PBS and then incubated with biotinylated anti-rabbit antibody (BA-1000-1.5, Vector Laboratories) for 30 minutes at room temperature. VECTASTAIN ELITE ABC-HRP Reagent (Vector Laboratories) was then added to tumor sections for another 30 minutes at room temperature. Mayer’s hematoxylin (Sigma-Aldrich) was applied to the slides for counter-staining. Tumor slides were dehydrated and sealed with coverslips. Ten fields per tumor slide were randomly selected and counted by an observer blinded to treatment at 40× magnification for the number of CD8^+^ T cells.

### Reagent information

See Supplemental Table 3 for antibody clones, catalog numbers, and mouse strains.

### Statistics

Experiments were designed such that littermate controls were used for all experiments. For bar graphs, all data are presented as mean ± SEM. For comparison of time to tumor quintupling in tumor growth experiments, we performed a Kruskal-Wallis test (after all groups failed the Kolmogorow-Smirnov test for normality) on the time to tumor quintupling (days) for each sample stratified by treatment group. To identify differences between groups, we performed a pairwise Wilcoxon test. *P* ≤ 0.05 was considered significant. This was complemented by a Kaplan-Meier curve and a log-rank test to compare survival without tumor quintupling across treatment groups.

### Study approval

All animal studies were performed in accordance with protocols approved by the Duke University IACUC and adhered to the *Guide for the Care and Use of Laboratory Animals* (National Academies Press, 2011).

### Data availability

All sequencing data generated for this manuscript have been deposited in publicly accessible databases. The p53/MCA bulk tumor RNA-Seq data generated in this study are available in the NCBI Gene Expression Omnibus (GEO) database under the accession no. GSE252213. The p53/MCA scRNA-Seq data generated in this study are available in the NCBI Gene Expression Omnibus (GEO) database under the accession no. GSE252143. The mass cytometry data generated in this study are available in the flowrepository.org database under the ID code FR-FCM-Z74J. Code for scRNA-Seq analysis in the manuscript can be found at https://github.com/DukeChangSu/CpG-RT-single-cell-RNAseq-analysis-code (commit ID = 6d3d671). Values for all data points in graphs are reported in the Supporting Data Values file. Source data are provided with the paper.

## Author contributions

CS and DGK conceptualized the project. CS, CLK, AJW, EJM, YMM, and DGK developed the methodology. CS, CLK, AS, VMP, RDHB, KER, JLM, SRS, and EJM conducted statistical analysis. CS, CLK, MP, LL, NTW, YM, WF, BP, ALL, and JEH performed the investigation. CS and DGK wrote the original draft of the manuscript. CS, AS, VMP, MLS, AJW, EJM, YMM, MP, and DGK reviewed and edited the manuscript. CS was responsible for visualization. DGK supervised the project. CS and CLK are co–first authors. CS is listed first due to her writing contributions.

## Supplementary Material

Supplemental data

Supplemental table 1

Supplemental table 2

Supplemental table 3

Supporting data values

## Version 1 07/22/2024

Electronic publication
