# Supplementary material for: Enhancing radiotherapy response via intratumoral injection of a TLR9 agonist in autochthonous murine sarcomas
Source: JCI Insight. 2024 Jun 13;9(14):e178767. doi: 10.1172/jci.insight.178767 (PMC11383182; doi:10.1172/jci.insight.178767)
Supplement: Supplemental data [file jciinsight-9-178767-s179.pdf]

Cytotoxicity study for CpG effect on tumor cells

Generation of sarcoma cell lines

We generated individual cell lines from three murine autochthonous p53/MCA sarcomas as described above. Tumors were dissected from the limb and dissociated by shaking for 45 min at 37°C in an enzyme mix from the tumor dissociation kit (Miltenyi Biotec, 130096730). Cell suspension was then strained through a 40 µm filter, washed in PBS, and plated for culture. Cell lines were maintained *in vitro* for 5 passages before coincubation with CpG in 96-well plates.

In vitro tumor proliferation assay with CpG through IncuCyte

$1x10^4$ p53/MCA sarcoma cells were resuspended in 100µL of complete cell culture media and added to each well of a 96-well plate. After cells adhere to the bottoms of the plate overnight, 100µl of 0.01µg/µl, 0.1µg/µl, and 1µg/µl of CpG and 0.9ul of caspase-3/7 dye (Sartorius, 4440) were added to the well for 72hrs incubation in the SpectraMax M5 and Lmax microplate reader. Images of the sarcoma cells acquired by the microplate reader were analyzed by the Incucyte® Software and plotted using GraphPad Prism.

Analysis of interactions between myeloid cells and T cells

To investigate cellular interactions between myeloid cells and T cells, we utilized the CellChat R package (version 2.1.2) to predict ligand-receptor signaling between monocytes, macrophages, dendritic cells, and T cells using single cell RNA-seq. Significantly enriched ligand-receptor pairs in control and CpG plus RT treated tumors from the CellChat mouse database were identified using the

‘identifyOverExpressedGenes,’ ‘identifyOverExpressedInteractions,’ and ‘computeCommunProb’ functions and visualized using the ‘netVisual_circle’ and ‘netVisual_bubble’ functions with default parameters. Subsequently, the control and CpG plus RT CellChat objects were merged using the ‘mergeCellChat’ function for comparison (1, 2).

Supplemental Methods References:

1. Jin S, et al. Inference and analysis of cell-cell communication using CellChat. *Nat Commun*. 2021;12(1):1088.

2. Jin S, Plikus MV, Nie Q. CellChat for systematic analysis of cell-cell communication from single-cell and spatially resolved transcriptomics. *bioRxiv*. 2023;2023.11.05.565674.

Supplementary Figure 1

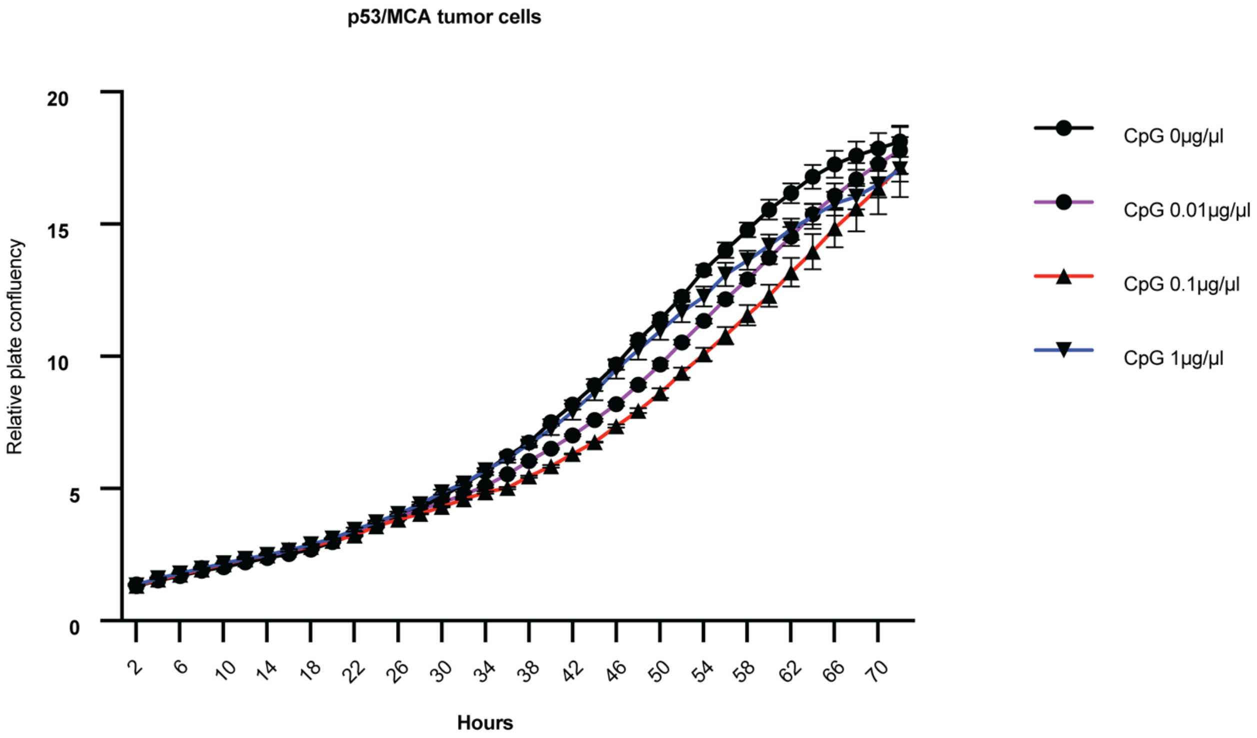

Supplementary Fig 1. **IncuCyte Live-Cell assay showed no direct effect on cell proliferation after co-incubation of p53/MCA sarcoma cells with titrated concentrations of CpG.**

IncuCyte cell proliferation assay of p53/MCA sarcomas incubated with four serial dilutions of CpG. The data is representative of three biological replicates.

Supplementary Figure 2

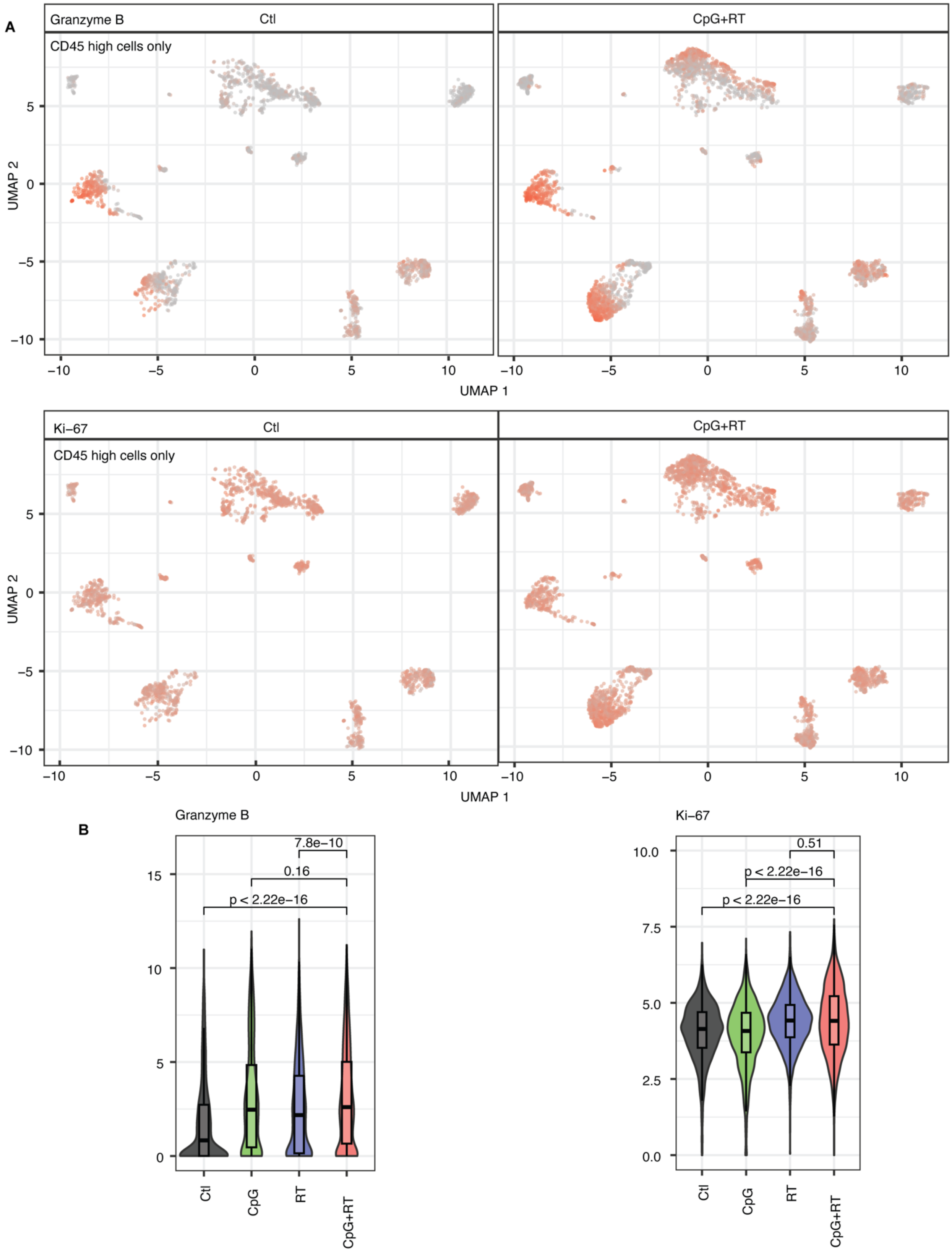

Supplementary Fig 2. CyTOF demonstrates enhanced Granzyme B and Ki-67 expression in CD8^{+} T cells after treatment with CpG+RT.

A. UMAP plot of CyTOF data clustering for CD45hi cells with Granzyme B and Ki67 expression colored by the ion intensity. In CD8+ cells, Granzyme B and Ki-67 were highly co-expressed (spearman coefficient = 0.36, p <= 0.0001). **B.** Violin/boxplot of granzyme B and Ki-67 ion intensity in CD45 high cells across all treatment groups. A Wilcox rank sum test was used for statistical analysis.

Supplementary Figure 3

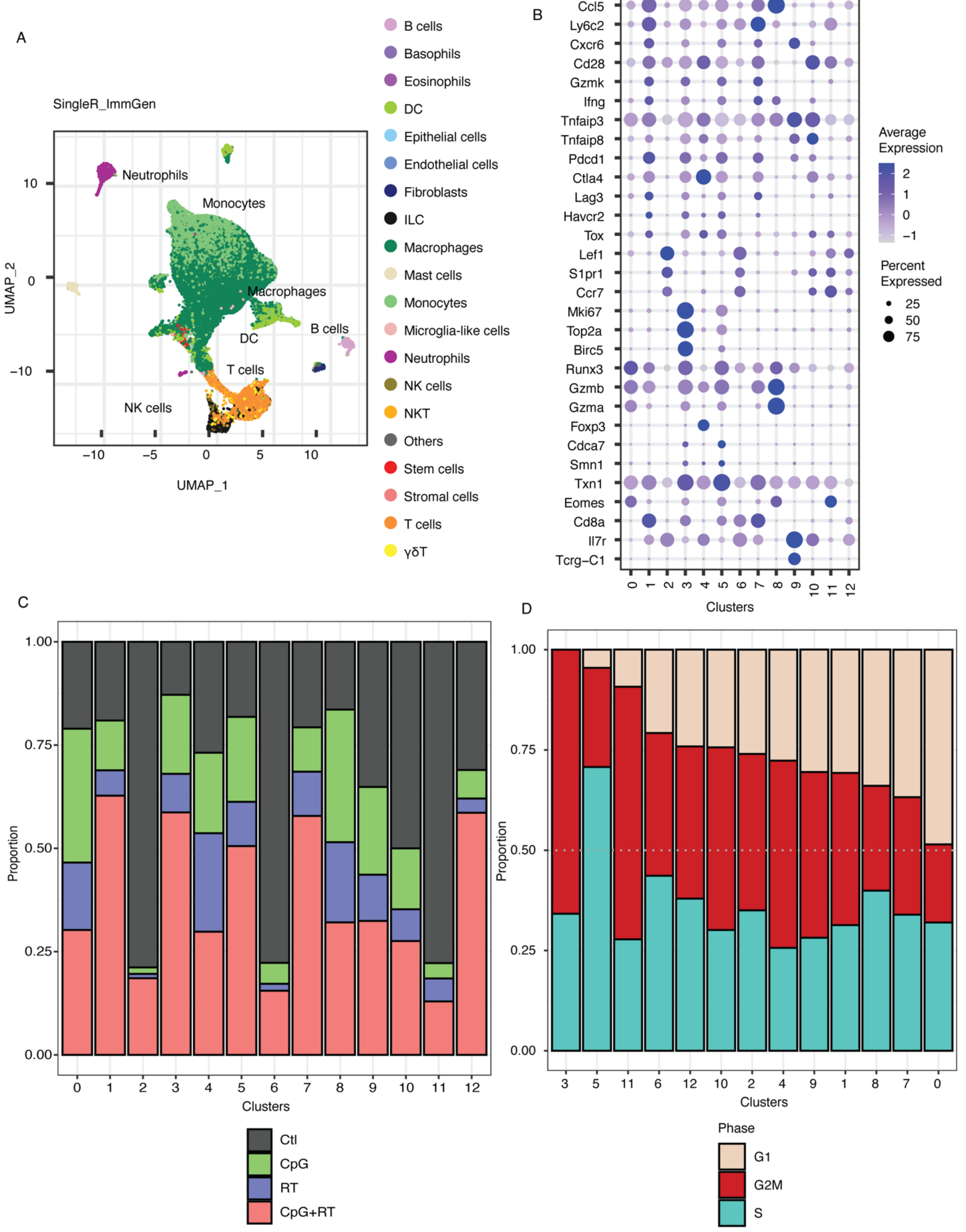

Supplementary Fig 3. Single cell RNA-seq shows increased CD8$^+$ T cells infiltration in the tumor area after CpG+RT that is highly proliferative and activated.

A. UMAP plot of scRNA-seq clustering for all CD45hi cells from all tumors and treatment groups. **B.** Bubble plot of characterizing genes in each of the T cell subclusters. **C**. Proportion of T cells contributed from each treatment group. **D**. Phases of cell cycle of clusters of different T cells and NK cells.

Supplementary Figure 4

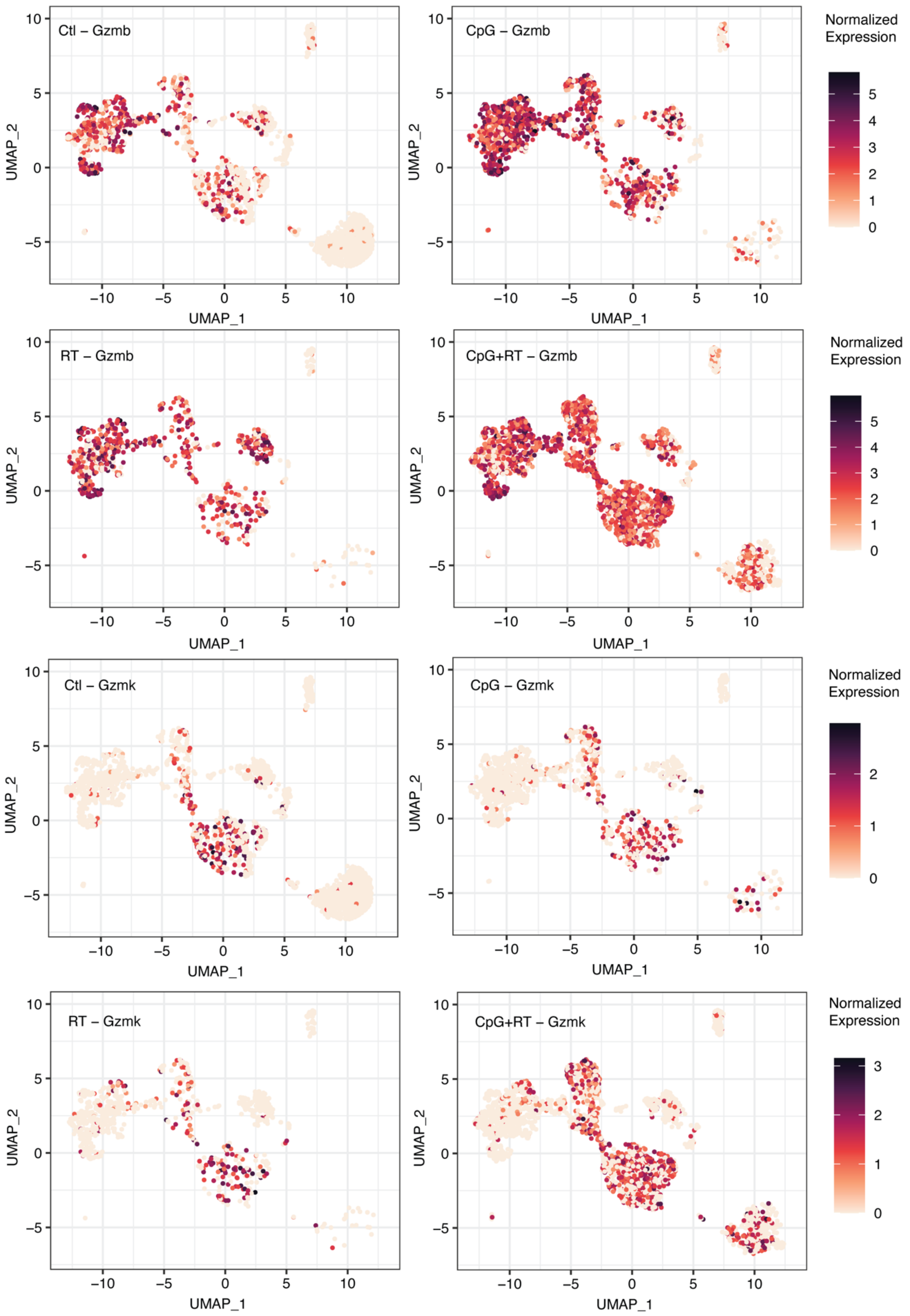

Supplementary Fig 4. Single cell RNA-seq shows elevated expression of Granzyme B, Granzyme K, and IFNγ in CD8^{+} T cells after treatment with with CpG+RT.

UMAP plots of NK cells and T cells with Granzyme B, Granzyme K, and IFNγ expressing cells highlighted before and after CpG+RT treatments.

Supplementary Figure 5

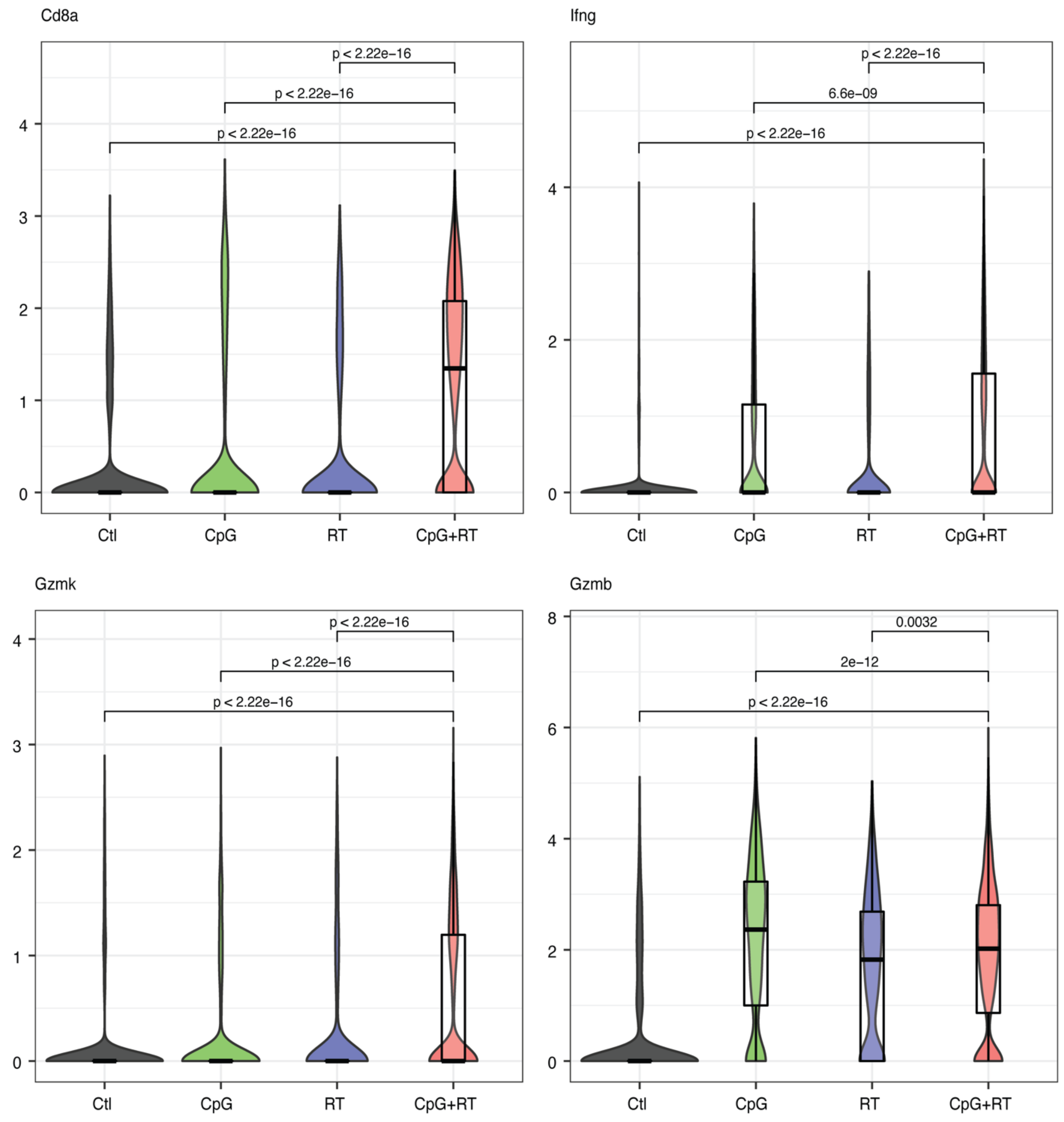

Supplementary Fig 5. Statistical analysis and violin plots of CD8, IFNγ, Granzyme B, and Granzyme K mRNA levels in single cell RNA-seq data across different treatment groups.
Normalized count of Cd8a, IFNγ, Gzmk, and Gzmb in single-cell transcriptomic data stratified by treatment group. P values were calculated using two-sided Wilcoxon tests.

Supplementary Figure 6

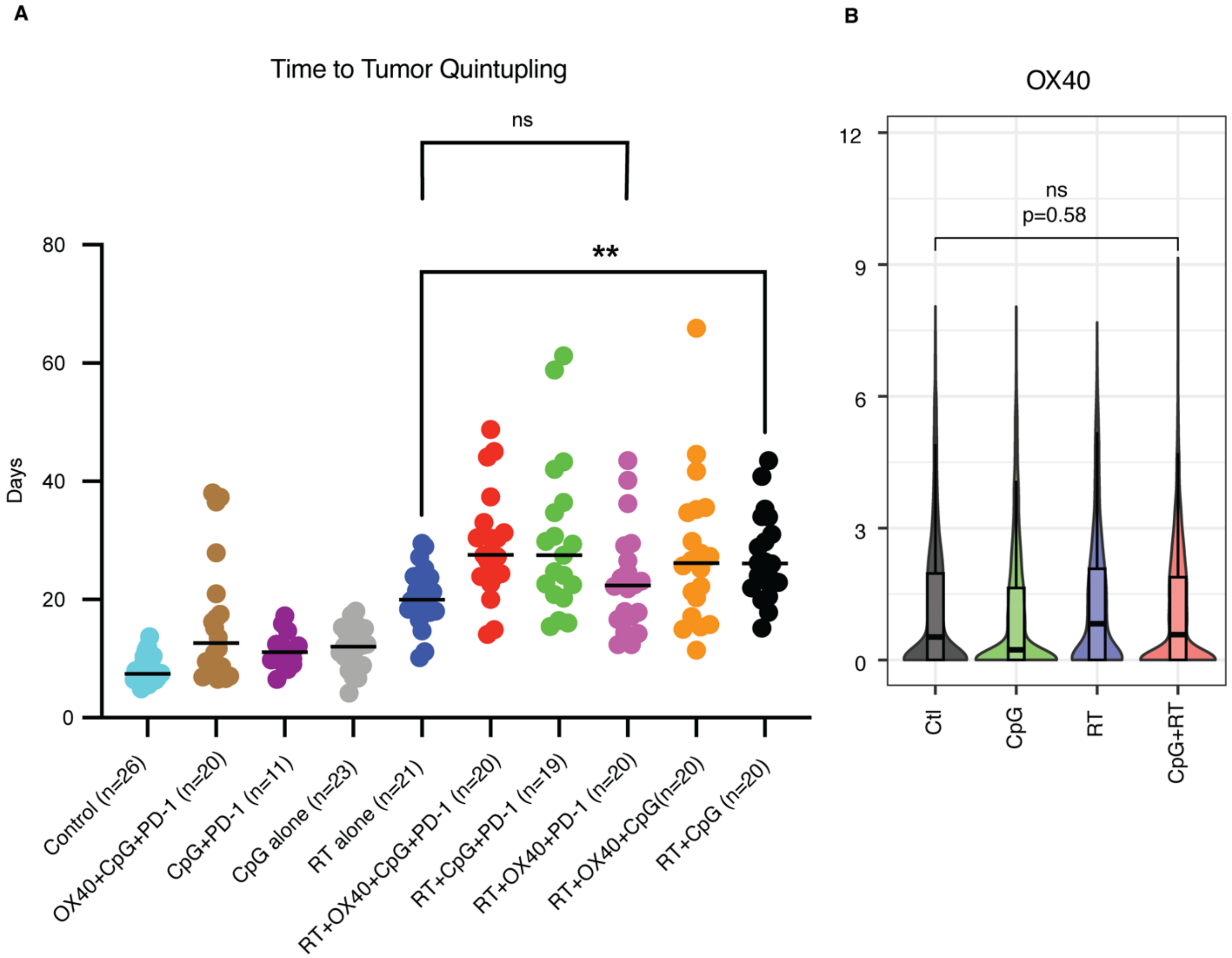

Supplementary Fig 6. Time to tumor quintupling for p53/MCA sarcomas treated with different combination therapies.

A. p53/MCA sarcomas were treated with anti-OX40 or vehicle control, anti-PD1 or IgG2a isotype control, CpG ODN or control GpC dinucleotides, and 0 or 20 Gy when tumors reached >70 mm^3. Kruskal-Wallis Test was used for the group comparison, while the Wilcoxon test was selected for the pair-wise comparisons. * shows the significance of the P Value (**: p<=0.01, ***: p <= 0.001, ****: p <= 0.0001). **B.** Violin/boxplot of OX40 protein intensity in CD45 high cells across all treatment groups through CyTOF. P value was calculated with Wilcoxon rank sum test.

Supplementary Figure 7

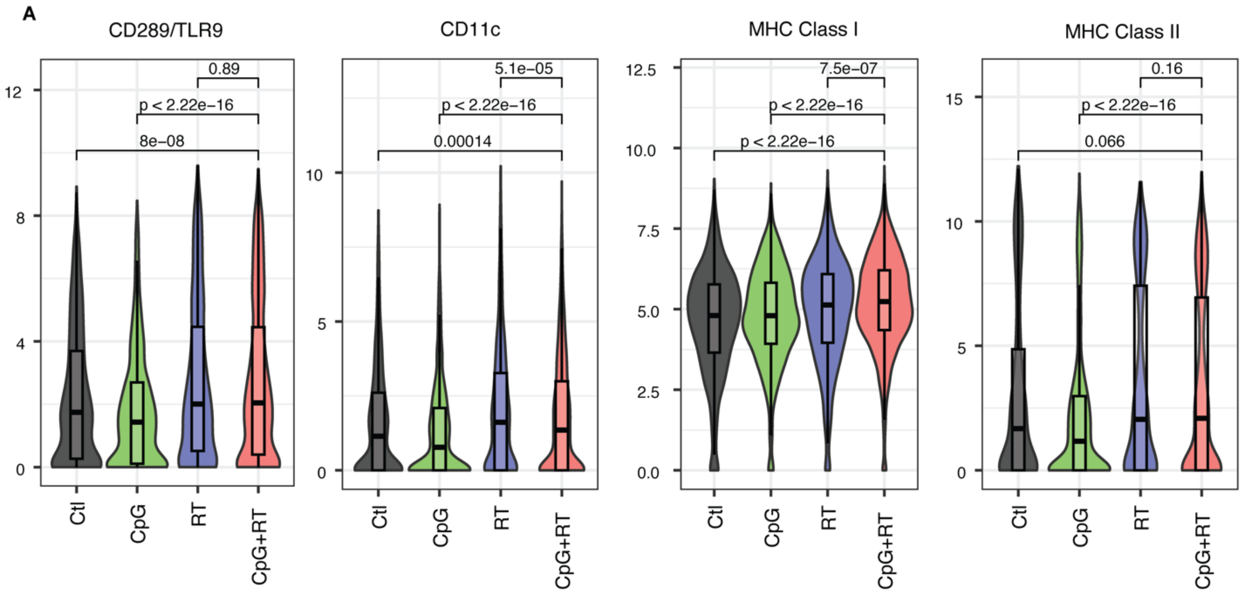

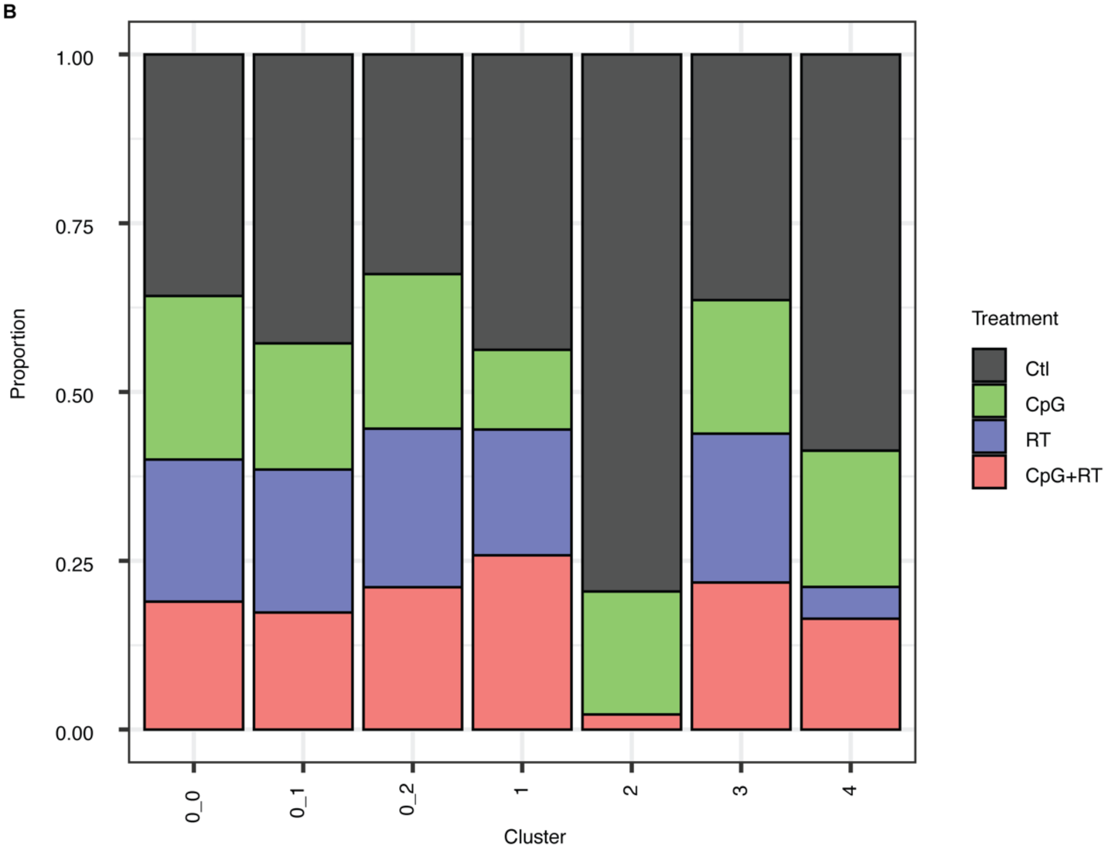

Supplementary Fig 7. Treatment with CpG+RT promotes intratumoral dendritic cell remodeling.

A. Violin/boxplot of CD289/TLR9, CD11c, MHC Class I, and MHC Class II protein intensity in CD45 high cells across all treatment groups through CyTOF. A Wilcox rank sum test was used for statistical analysis. **B.** Proportion of DCs contributed from each treatment group.

Supplementary Figure 8

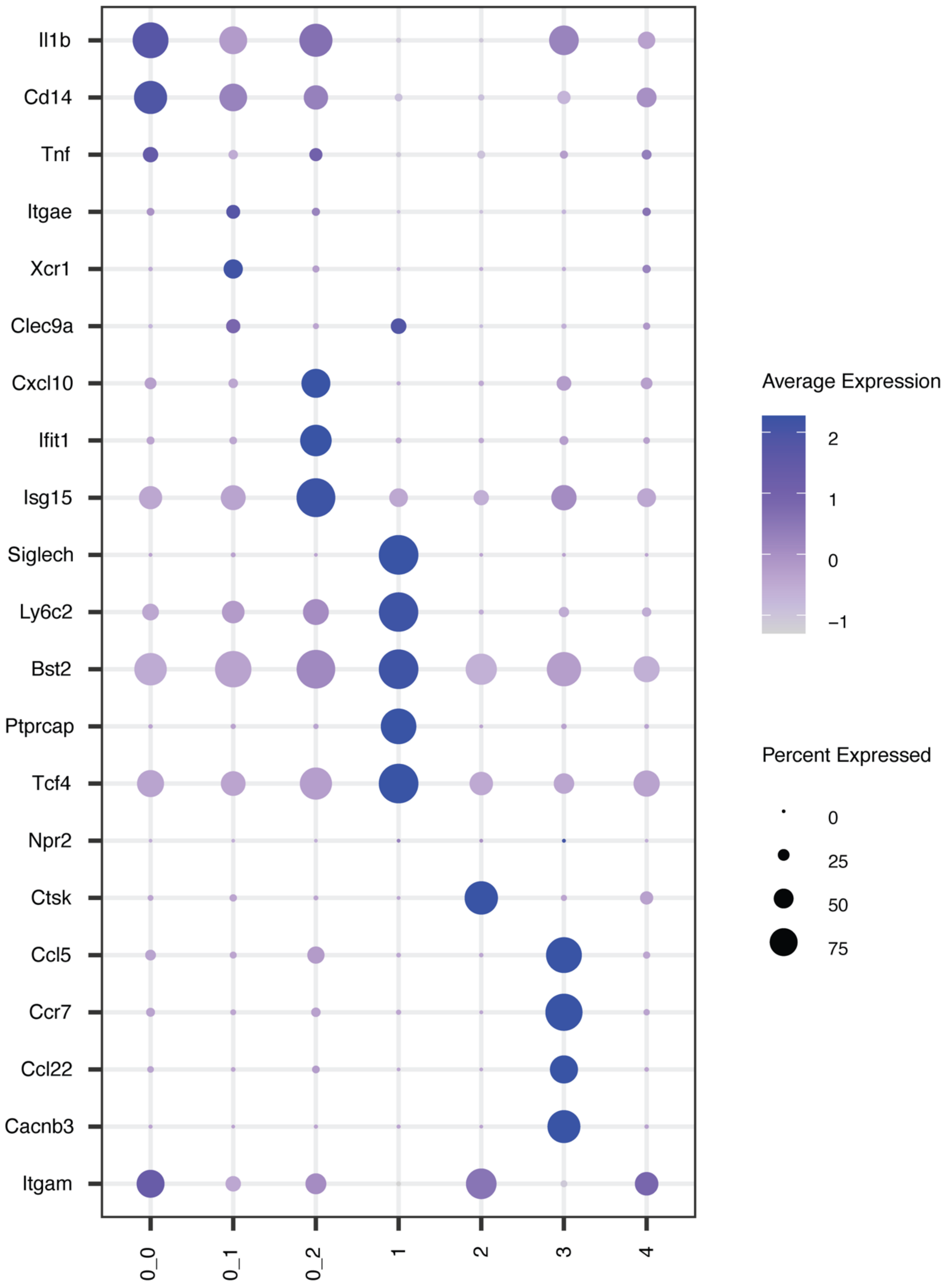

Supplementary Fig 8. Bubble plot of characterizing genes in each of the DC subclusters.

Supplementary Figure 9

A

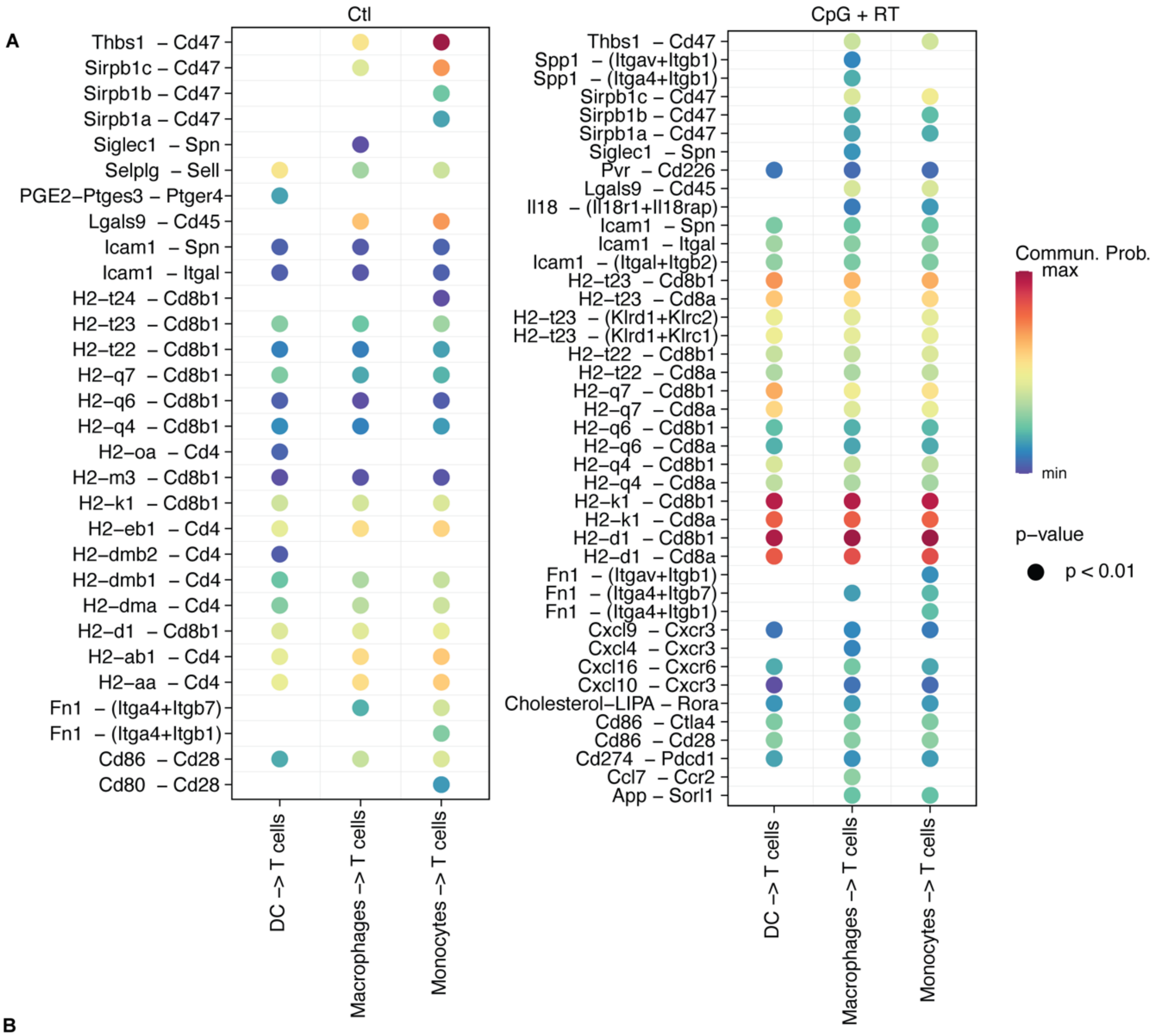

B

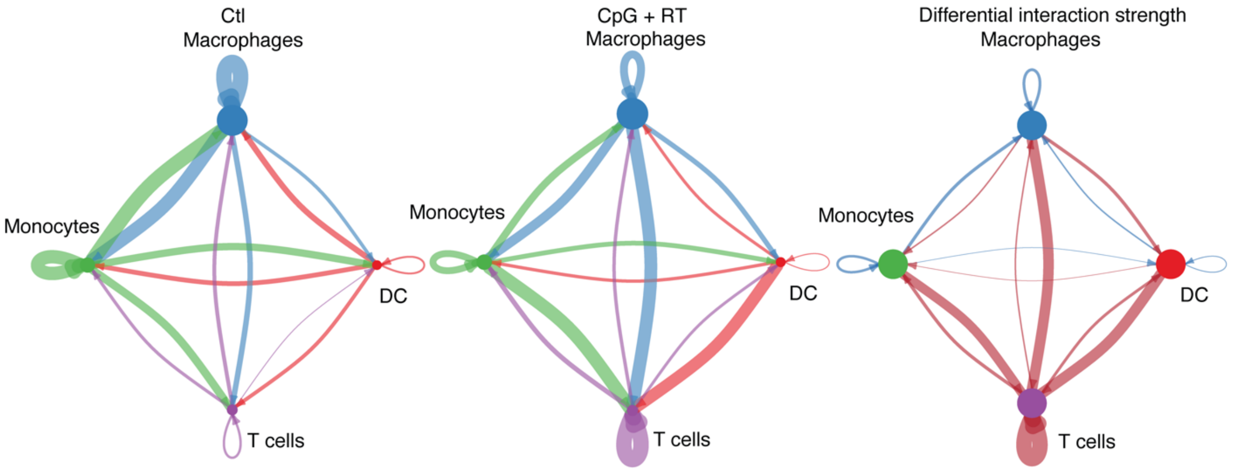

Supplementary Fig 9. Cell Chat analysis between myeloid cells and T cells from scRNA-seq data.

A. All the significant ligand-receptor pairs that contribute to the signaling from DC, macrophages, and monocytes to T cells. The dot color and size represent the calculated communication probability and *p*-values. *p*-values are computed from the one-sided permutation test. **B**. Network Plots that represent the differential interaction strength of ligand-receptor pairs between monocytes, macrophages, DCs, and T cells populations in mice with p53/MCA sarcomas received control GpC dinucleotides with 0 Gy (n=5) and CpG ODN with 20 Gy (n=5). The edge width is proportional to the interaction strength of ligand-receptor pairs.

Supplementary Figure 10

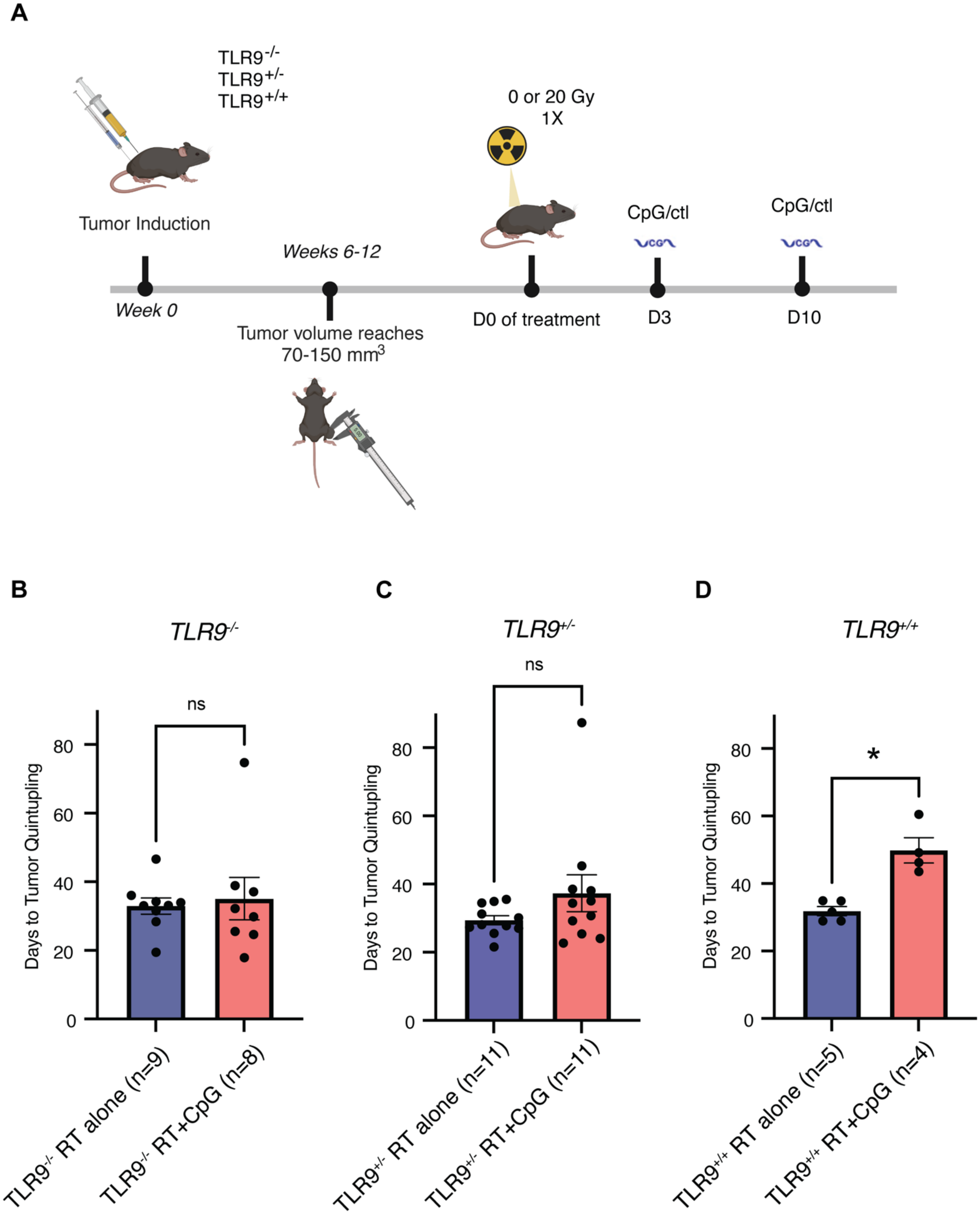

Supplementary Fig 10. TLR9 receptor mediates the anti-tumor effects of the combination treatment CpG+RT.

A. Primary sarcoma initiation by intramuscular injection of Adeno-Cas9-sgp53 and MCA. Autochthonous sarcoma develops at the injection site about 6-12 weeks after induction. Mice were treated with control GpC dinucleotides with 20 Gy or CpG ODN with 20 Gy when tumors reached >70 mm^3. **B.** Homozygous KO mice (*TLR9*$^{-/-}$) with p53/MCA sarcomas received control GpC dinucleotides with 20 Gy (black, *n* = 9) or CpG ODN with 20 Gy (red, *n* = 8). The figure shows time to tumor quintupling (days). **C.** Heterozygous mice (*TLR9*$^{+/-}$) with p53/MCA sarcomas received control GpC dinucleotides with 20 Gy (black, *n* = 11) or CpG ODN with 20 Gy (red, *n* = 11). **D.** Homozygous WT mice (*TLR9*$^{+/+}$) with p53/MCA sarcomas received control GpC dinucleotides with 20 Gy (black, *n* = 5) or CpG ODN with 20 Gy (red, *n* = 4). Figure shows time to tumor quintupling (days). Wilcoxon Rank Sum Test was used to compare differences between two treatment groups with the same genotype. * shows the significance of the P Value (ns: p > 0.05, *: p <= 0.05).
